# Unifying microorganisms and macrograzers in intertidal rocky shore ecological networks

**DOI:** 10.1002/ecy.70275

**Published:** 2026-01-14

**Authors:** Clara Arboleda‐Baena, Claudia Belén Pareja, Javiera Poblete, Eric L. Berlow, Hugo Sarmento, Ramiro Logares, Rodrigo De la Iglesia, Sergio A. Navarrete

**Affiliations:** ^1^ German Centre for Integrative Biodiversity Research (iDiv), Synthesis Centre (sDiv) Leipzig University Leipzig Germany; ^2^ Laboratory of Microbial Processes & Biodiversity, Hidrobiology Departament Universidade Federal de São Carlos São Carlos Brazil; ^3^ Estación Costera de Investigaciones Marinas (ECIM) Pontificia Universidad Católica de Chile Las Cruces Chile; ^4^ Laboratorio de Microbiología Marina, Facultad de Ciencias Biológicas Pontificia Universidad Católica de Chile Santiago Chile; ^5^ Vibrant Data Labs Berkeley California USA; ^6^ Institute of Marine Sciences (ICM), CSIC Barcelona Spain; ^7^ Marine Energy Research & Innovation Center (MERIC) Santiago Chile; ^8^ Núcleo Milenio para la Ecología y la Conservación de los Ecosistemas de Arrecifes Mesofóticos Templados (NUTME) Pontificia Universidad Católica de Chile Santiago Chile; ^9^ Center for Applied Ecology and Sustainability (CAPES) Pontificia Universidad Católica de Chile Santiago Chile; ^10^ Centro de Oceanografía Copas Coastal Universidad de Concepción Concepción Chile

**Keywords:** bacteria, epilithic biofilms, interaction strength, mollusk grazers, periphyton, trophic and non‐trophic interactions

## Abstract

Over the past decades, our understanding of the vital role microbes play in ecosystem processes has greatly expanded. However, we still have limited knowledge about how microbial communities interact with larger organisms. Many existing representations of microbial interactions are based on co‐occurrence patterns, which do not provide clear insights into trophic or non‐trophic relationships. In this study, we untangled trophic and non‐trophic interactions between macroscopic and microscopic organisms on a marine rocky shore. Five abundant mollusk grazers were selected, and their consumptive (grazing) and nonconsumptive (grazer pedal mucus) interactions with bacteria in biofilms were measured using 16S rRNA‐gene amplicon sequencing. While no significant effects on a commonly used measure of biofilm grazing (chlorophyll *a* concentration) were observed, detailed image analysis revealed that all grazers had a detrimental impact on biofilm cover. Moreover, different grazers exhibited distinct effects on various bacterial groups. Members of the Alteromonadaceae, Burkholderiaceae, Flavobacteriaceae, Halieaceae, Phycisphaeraceae, Rhodobacteraceae, Rickettsiaceae, Saprospiraceae, and Vibrionaceae families experienced positive trophic effects from specific grazers. In contrast, members of the Flavobacteriaceae, Pirellulaceae, Rhodobacteraceae, Rubritaleaceae, and Saprospiraceae families were negatively affected by trophic interactions with other grazers. Some members of the Gammaproteobacteria, Flavobacteriaceae, Ilumatobacteraceae, Pirellulaceae, Rickettsiales, Rhodobacteraceae, and Rubritaleaceae families exhibited non‐trophic positive interactions with specific grazers. Meanwhile, members of the Family DEV007 (Verrucomicrobiales), Flavobacteriaceae, Ilumatobacteraceae, Legionellaceae, Rickettsiales, Rhodobacteraceae, Saprospiraceae, and Xanthobacteraceae families exhibited non‐trophic negative interactions with particular grazers. Both trophic and non‐trophic interactions shift the microbial community toward enhanced recycling, energy efficiency, and stress resilience. Grazer activity, through biomass removal and exudates like pedal mucus, reduces photosynthetic groups like diatoms, halting dimethylsulfoniopropionate (DMSP) production and negatively impacting sulfur‐cycling bacteria and associated parasites. This research complements the ecological network of the intertidal rocky shore in central Chile and represents the first attempt to construct an interaction network between macroorganisms and bacteria. It reveals that the strength of trophic and non‐trophic interactions varies depending on the grazer and bacterial group involved. While some bacterial groups responded broadly, others showed specialized responses to specific macroorganisms. Overall, this study highlights the potential for integrating microbes into ecological networks, offering valuable insights methodologies for quantifying interactions across domains.

## INTRODUCTION

Organisms in ecosystems engage in complex interactions with multiple species, creating intricate networks where changes in one species can affect seemingly unrelated species in unpredictable ways (Yodzis, 2001). Beyond trophic interactions (TIs), where organisms obtain energy by consuming biomass (Lindeman, 1942), there are numerous non‐trophic interactions (NTIs) with diverse effects, including positive ones like commensalism, facilitation, and mutualism, as well as negative ones like inhibition and interference (Kéfi et al., 2012). Network representations, where co‐occurring species are nodes and a single type or multiple types of interactions (multiplex networks) as vertices or links, offer a framework to study the structure and dynamics of complex ecological networks (Bascompte, 2010). Substantial progress has been made in recent years, enabling realistic visualization and characterization of these networks (Kéfi et al., 2016; Sander et al., 2015, 2017) and modeling their dynamics (Rebolledo et al., 2019; Ryser et al., 2021; Valdovinos, 2019).

**FIGURE 1:**
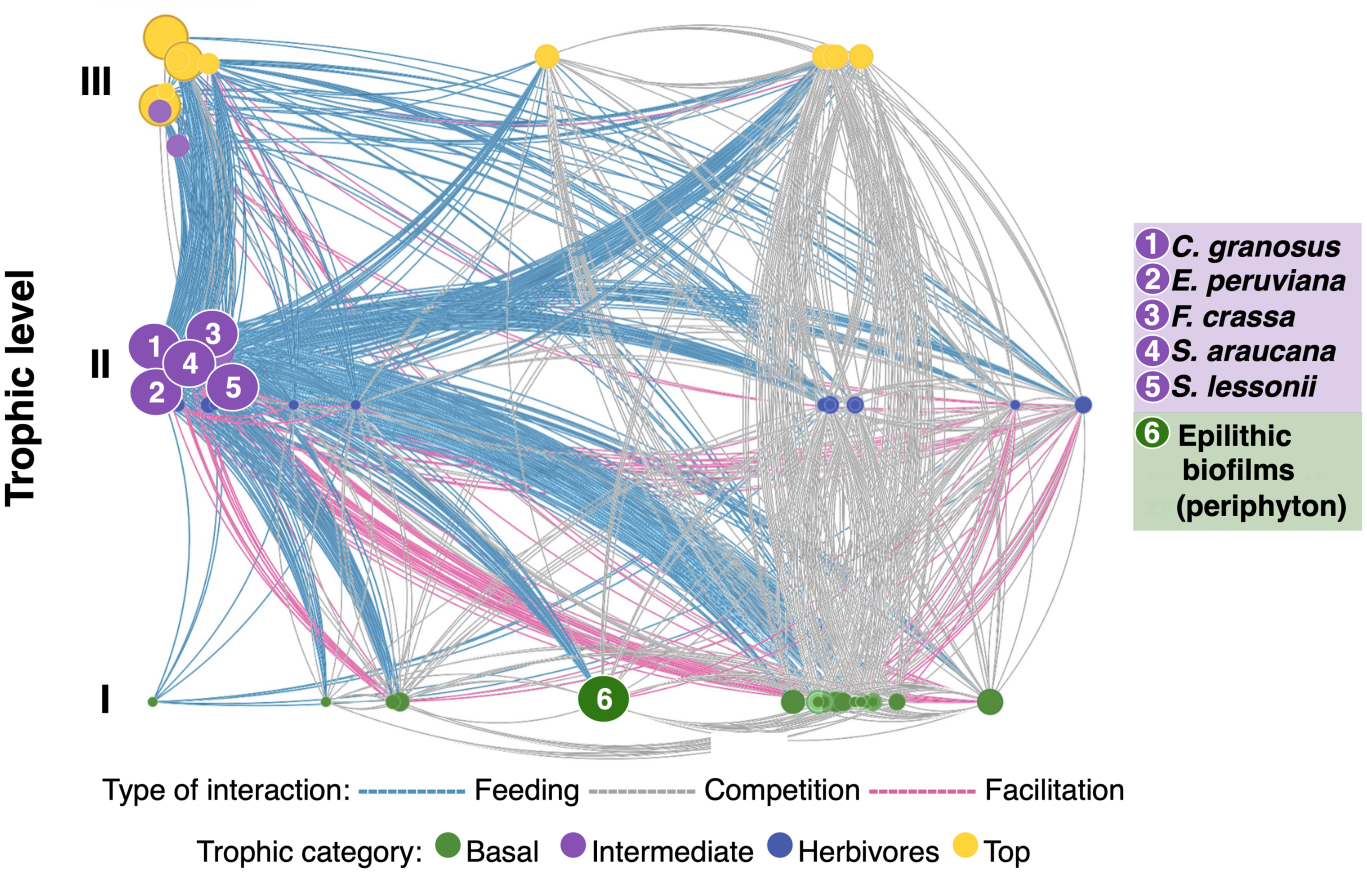
Ecological network of known species interactions in the Chilean marine rocky shore ecosystem. Nodes represent species, and the links depict three types of interactions: feeding (blue), competition (gray), and facilitation (pink). The node's color is the trophic category: green—basal, purple—intermediate, blue—herbivores, yellow—top. Interactions between the most abundant grazers and the epilithic biofilm are highlighted. Image adapted from Vibrant Data Labs.

Coastal marine ecosystems have provided some of the most detailed ecological networks due to extensive experimental research on TIs and NTIs (Kéfi et al., 2015, 2016; Sander et al., 2015, 2017). However, a common weakness in well‐documented marine and terrestrial food webs (Digel et al., 2014; Lafferty et al., 2008; Link, 2002), as well as multiplex ecological networks (Costa et al., 2020; Hale et al., 2020), is the neglect or misrepresentation of co‐occurring microorganisms that interact with macroorganisms (Sechi et al., 2015). Microorganisms are the most abundant and diverse organisms on Earth (Locey & Lennon, 2016). They are essential for regulating biogeochemical cycles (Gasol & Kirchman, 2018), providing materials and energy to higher trophic levels (Castenholz, 1961; Thompson et al., 2000). While our understanding of microorganisms at the ecosystem level is well established, we remain profoundly ignorant about their interactions with macroorganisms at the community level (Bjorbækmo et al., 2020). Despite extensive research on microbiomes within macroscopic organisms, our understanding of the microbial world has only recently shifted from viewing it as a separate, non‐interacting entity (Miller et al., 2018; Robinson et al., 2024; Sarkar et al., 2020). Neglecting these interactions hinders our ability to realistically capture ecological network dynamics and understand the functioning of complex ecological systems (Koskella et al., 2017). To approach this problem, we used one of the most resolved ecological networks as a study model, the intertidal rocky shore of central Chile (Kéfi et al., 2015, 2016). Our study aimed to uncover the trophic and non‐trophic connections between the prevalent intertidal mollusk grazers (*Chiton granosus*, *Echinolittorina peruviana*, *Fissurella crassa*, *Scurria araucana*, and *Siphonaria lessonii*) and the bacterial components of the co‐occurring epilithic biofilms (periphyton) (Figure 1; Arboleda‐Baena et al., 2024). Here, we use epilithic biofilm as a general term for communities comprising organisms from all three domains of life that are bound together by extracellular polymeric substances and grow on rock surfaces (Schuster et al., 2019), whereas the term periphyton is used specifically to refer to the phototrophic fraction of these biofilms.

In TIs, epilithic biofilms are essential for the primary productivity of coastal ecosystems (Bustamante et al., 1995; Jenkins et al., 2001; Thompson et al., 2000, p. 202). On many shores, they are the primary food resource for many benthic grazers (Castenholz, 1961; Christofoletti et al., 2011; Thompson et al., 2000). The molluscan grazers feed by scraping rock surfaces, removing algae, invertebrate settlers, and microorganisms to varying extents depending on the species and their ecological and morphological traits (Aguilera & Navarrete, 2012a; Christofoletti et al., 2011; Jenkins et al., 2001; Santelices et al., 1986; Underwood, 1984). The most common method for analyzing grazer effects on epilithic biofilms (periphyton) is by measuring chlorophyll *a* content or concentration as an integrated indicator of biofilm biomass (Jenkins et al., 2001; Underwood, 1984; Williams et al., 2000). While this integrated measure is important for understanding ecosystem‐level functions, it only captures the photosynthetic components of the biofilm community. Consequently, this approach misrepresents the potentially rich and distinctive effects of macro‐grazers on specific biofilm taxa and patterns of community structure.

Meanwhile, NTIs can occur as animals move above the rocks, searching for food, escaping from predators, seeking shelter, and protecting from waves. During these activities, the pedal mucus microbiota of these animals could come into contact with the microbial communities present in the epilithic biofilms. Interactions involving grazers and epilithic biofilms have been shown to have both positive and negative effects, mediated by the grazers' pedal mucus (Arboleda‐Baena et al., 2022; Davies et al., 1990).

Indeed, the resistance and resilience in integrated measures of biofilm communities may result from different microbial groups responding variably to herbivore‐specific grazing. Many significant changes in community structure could go unnoticed if, for instance, photosynthetic biomass is dominated by a few abundant groups. Therefore, it is critical to advance and apply new methodologies that allow us to address more specific questions about the interaction between macro‐grazers and biofilms. For example: Are the recovery dynamics (resilience) of epilithic biofilms different for each grazer species? How does biofilm community composition change in response to different grazers? Are TIs and NTIs similar across grazers, or are NTIs more general or more specific than TIs within the biofilm community? Are certain microbial organisms more susceptible and therefore more significantly affected by TIs than NTIs from different grazers? These are broad questions for which we currently lack answers. Examining the combined effect of grazers on integrated biofilm variables alone will not allow us to disentangle the web of macro–micro interactions. To address this, we have developed an experimental approach to quantify the strength of both TIs and NTIs (Aguilera & Navarrete, 2012a; Berlow et al., 1999; Paine, 1992; Wootton, 1997). This approach is repeatable and can provide comparative measures across different grazers and biofilm communities.

We begin by conducting traditional experiments to quantify the impact of grazers on integrated measures of epilithic biofilm abundance, such as chlorophyll *a* content and biofilm cover, using five different intertidal grazer species. Next, we perform controlled experiments using molecular techniques to investigate whether TIs (consumptive) between grazers and biofilms have a greater influence on the bacterial community than NTIs (mediated through pedal mucus), and to determine which biofilm components are affected. We selected five model intertidal grazer species from distantly related mollusk taxa, each with different morphological traits (e.g., body size) and feeding modes (e.g., radula structure), to examine their effects on the bacterial components of the biofilm community. This approach will allow us to unravel and quantify the network of interactions between specific grazers and distinct bacterial groups.

Thus, in this study, we shift from analyzing epilithic biofilm community dynamics on a broader scale (e.g., integrated measures of abundance such as chlorophyll *a* content and biofilm cover) to investigating the interaction strength between specific grazers and distinct bacterial groups. This approach represents the first attempt to disentangle the interaction strength between macroorganisms and bacteria within the biofilm. However, it did not include all grazer species co‐occurring in the intertidal zone, nor did the paired experiments account for interactions between grazer species, which are known to occur (Aguilera et al., 2013). Additionally, other higher order interactions, such as nonlinear density effects, were not considered.

## MATERIALS AND METHODS

### Study site and mollusk grazer assemblage

The study was conducted at the Estación Costera de Investigaciones Marinas (ECIM) of Pontificia Universidad Católica de Chile, located in Las Cruces, Chile (33°30′ S, 71°38′ W). We chose five of the most abundant grazer species in terms of total biomass (Arboleda‐Baena et al., 2022), one Polyplacophoran, the chiton *Chiton granosus* (Frembly 1828) Family Chitonidae, and four Gastropods: the Littorinid *Echinolittorina peruviana* (Lamarck 1822) Family Littorinidae, the keyhole limpet *Fissurella crassa* (Lamarck 1822) Family Fissurellidae, the scurrinid limpet *Scurria araucana* (d'Orbigny 1839) Family Lottiidae, and the pulmonate limpet *Siphonaria lessonii* (Blainville 1827) Family Siphonariidae (Aguilera et al., 2013; Espoz et al., 2004). These omnivores scrape rock surfaces, primarily removing periphyton (epilithic biofilm), ephemeral algae, and newly established invertebrates (Aguilera & Navarrete, 2012a; Camus, 2008). Field study approval number ID Protocol: 170829006, by the Comité Institucional de Seguridad en Investigación of the Pontificia Universidad Católica de Chile.

### Grazer and epilithic biofilm sampling

First, field rocks were collected and cut into coupons of 3 × 8 × 2 cm using a COCH Bridge saw machine to prevent overheating and mineral modification. The coupons were then cleaned with deionized water, dried, and kept at room temperature until introduced into the experimental aquaria. To establish epilithic biofilm communities, the rock coupons were placed in aquaria with circulating seawater sourced from the same location and provided with continuous aeration between September and October 2018.

Second, we collected 50 individuals of each grazer species from gently sloping wave‐exposed platforms near ECIM. The collection occurred during nocturnal low tides to avoid foot damage (Aguilera & Navarrete, 2012a). The collected animals were transported in coolers to the laboratory and then, to reduce animal stress, acclimatized for a week in separate aquaria with circulating seawater and constant aeration. During this period, the grazers were exclusively fed with epilithic biofilm provided in the circulating seawater. Then, to minimize fecal contamination before the experiment, the grazers underwent a 2‐day cleaning period in an aquarium with constant aeration and 0.2‐μm filtered seawater (from the same location). During this period, the animals starved, reduced fecal production, and avoided adverse locomotory and metabolic effects (Calow, 1974). On the third day, reduced motility was observed, indicating the effective prevention of locomotory and metabolic disturbances during the cleaning period (data not shown). The filtered water was replaced every 2–6 h to minimize ammonium concentration and prevent biofilm formation associated with animals' feces. This cleaning period also helped remove incidental microorganisms on the animal foot that do not maintain populations in the pedal mucus. The motility and behavior of the animals were monitored throughout the acclimation period. Seawater temperature was maintained at 13 ± 2°C, the average SST during the experiments.

### Grazer effects on integrated measures of epilithic biofilm abundance (chlorophyll *a* and cover)

To quantify grazing effects on total chlorophyll *a* content and cover of epilithic biofilm, we conducted a replicated laboratory experiment at ECIM. We measured the body size of each mollusk grazer and then calculated the average body size for each species (Appendix S1: Table S1) and classified them by their radula, from greater to lesser excavation capacity, in the following order (radula type in parenthesis): *C. granosus* (Steroglossa), *S. araucana* (Docoglossa), *S. lessonii* (Taenioglossa), *E. peruviana* (Taenioglossa), and *F. crassa* (Rhipidoglossa) (Reid, 2002; Steneck & Watling, 1982).

Ten grazers (*n* = 10) of each species were chosen randomly and placed in individual clean aquaria (14.3 × 14.3 × 12.5 cm) with 400‐mL filtered seawater to 0.22 μm (from the same location). Six grazing treatments (a–f) with 10 replicates each were implemented: (a) Control: rock coupon with epilithic biofilm without grazers, and (b–f) Rock coupons with epilithic biofilms plus one individual of either (b) *C. granosus*, (c) *E. peruviana*, (d) *F. crassa*, (e) *S. araucana*, (f) *S. lessonii*. Treatments were randomly assigned to the 60 experimental units (10 replicates per treatment). Every 3 h, the temperature was checked, and feces were removed with sterile pipettes. Filtered water was replaced every 6 h to reduce ammonium or other dissolved nutrients, prevent biofilm formation, and remove incidental microorganisms from the animal foot. The experiment lasted 24 h, and the animals were carefully extracted. Within 24 h, all 10 replicate rocks for each treatment were photographed, and the biofilm cover was analyzed using the software Image J with the protocol described in Protocols.io: https://doi.org/10.17504/protocols.io.e6nvw5epdvmk/v1 (Arboleda‐Baena, Pareja, et al., 2023). Biofilm total cover, expressed as a percentage of the rock coupon surface, provided an integrated estimate of grazers' total effect on autotroph and heterotroph biofilm components. Mean biofilm cover was compared among treatments with a Welch's ANOVA (six levels, fixed factor) due to deviations from homoscedasticity. Approximate normality was checked by visual inspection of residuals. To discern which treatment differed from others, a Games‐Howell nonparametric post hoc test was used, which performs well under deviation of homoscedasticity (Ruxton & Beauchamp, 2008). To assess effects only on autotrophs and compare with previous studies on epilithic algae (Aguilera et al., 2013; Valdivia et al., 2019; Williams et al., 2000, p. 200), three replicate rocks of each treatment from the prior experiment were randomly selected for chlorophyll *a* analyses. Rock coupons were individually sonicated (Morris et al., 1998) to remove all biofilm, which was recovered by filtration, preserved at 8°C in 50‐mL Falcon, and sent to the SEASON Environmental Analysis Laboratory for quantification using standard chlorophyll *a* method (American Public Health Association. et al., 1998). Results expressed as chlorophyll *a* concentration from the entire rock coupon surface and comparison among treatments were conducted with Welch's ANOVA (six levels, fixed factor) after inspection for normality and detection of slightly heterogeneous variance. To observe and qualitatively assess grazing effects on the biofilm community, we examined the grazed surface under an electron microscope. To this end, we chose one rock per treatment at random, cut a section of 1 × 1 × 1 cm with a diamond disk grinder, and immersed the piece for a few seconds in sterile seawater to remove the cut residue that could affect the biofilm. The rock sample was then fixed in 4% glutaraldehyde buffered with sodium cacodylate (0.1 M, pH 7.2) at 4°C. The coupon sections were scanned and photographed under a Hitachi TM3000 electron microscope at the Advanced Microscopy Laboratory (UMA) of the Pontificia Universidad Católica de Chile. To analyze the microbial composition of the epilithic biofilm at the experiment's onset, we obtained five replicates of the Control rock coupons (rock coupons with epilithic biofilm but without grazers). Each replicate was individually sonicated (Morris et al., 1998) to remove the biofilm, which was subsequently recovered by filtration using hydrophilic polyether sulfone filters with a pore size of 0.22 μm. The recovered biofilm was then preserved in liquid nitrogen at −196°C for subsequent DNA extraction and 16S rRNA‐gene sequencing. A diagram of the experiment is presented in Appendix S1: Figure S1.

### Assessing grazer's trophic and non‐trophic effects on epilithic biofilm community: Epilithic biofilm community analyses

To quantify the trophic (grazing) and non‐trophic (pedal mucus) interactions, a second experiment was conducted with 11 different treatments (a–k). Six treatments were implemented to examine the trophic effects (a–f), with 13 replicates each: (a) Control: epilithic biofilm rock control without grazers, and (b–f) One rock coupon with epilithic biofilm plus one individual of either (b) *C. granosus*, (c) *E. peruviana*, (d) *F. crassa*, (e) *S. araucana*, and (f) *S. lessonii*.

Simultaneously, we assessed the effect of pedal mucus by including five treatments (g–k) with 10 replicates each. In this case, rock coupons with epilithic biofilm were surrounded by a sterile transparent plastic mesh cage that impeded grazing on the surface. One individual of either (g) *C. granosus*, (h) *E. peruviana*, (i) *F. crassa*, (j) *S. araucana*, or (k) *S. lessonii* was included in each experimental aquarium. In these treatments, grazers moved on top of the protective mesh, and their pedal mucus was in contact with the filtered marine water on top of the rock coupons with epilithic biofilms. The pedal mucus microbiota came into contact with the epilithic biofilms, but the grazers could not move on top of the rock to graze it. See Appendix S1: Figure S1.

In this manner, we can examine: (1) Total grazer effects (T), in which treatments (b–f), where grazers feed in biofilm and pedal mucus is in contact with the rock's surface, are compared against controls without grazers; (2) NTI, in which treatments with only pedal mucus (g–k) are compared against the respective controls; and (3) TI, which is obtained by subtracting the respective pedal mucus effect from the total effect.

Treatments were randomly assigned to the 128 experimental units. Every 3 h, the temperature was checked, and feces were carefully removed. Filtered water was replaced every 6 h to reduce ammonium, prevent biofilm formation, and remove incidental microorganisms from the animal foot. The experiment lasted 24 h, and then the animals were carefully removed. Longer exposure was not possible because some grazers had already significantly reduced biofilm cover after 24 h and because we wanted to focus on the direct impacts of grazing on the resulting biofilm community. Thus, to analyze the effect of grazers on bacterial communities reassemblage, rocks were marked with graphite on the grazed zone, separating that zone from the rest of the epilithic biofilm community that remained on the rock. Rocks were transferred to new aquaria containing circulating seawater from the same location, along with constant aeration, for 10 days. This specific period was determined based on the observation of complete biofilm recovery on the rocks (data not shown). Then, after 10 days of recovery, 10 replicates of each treatment (a–k) were sampled with a sterile scalpel from control rocks, grazed areas of grazing treatment and pedal mucus treatment, placed on 0.22‐μm pore filters of hydrophilic polyether sulfone (Merck), and preserved in liquid nitrogen at −196°C for a subsequent DNA extraction and 16S rRNA‐gene sequencing. During the molecular analyses, we lost samples due to a poor‐quality Illumina sequencing run (see Appendix S1: Table S2).

Three replicates from each of the TI (grazing) treatments (treatments labeled as a–f) were subjected to analysis for total chlorophyll *a* concentration after a 10‐day recovery period, following the identical protocol described above. The chlorophyll *a* concentrations obtained from the grazed area of the rock coupon were standardized by area. Subsequently, to assess the statistical significance, Welch's ANOVA with six levels as a fixed factor was conducted after confirming the absence of homoscedasticity.

### DNA extraction and 16S rRNA‐gene sequencing

DNA extraction from filters was conducted with the Phenol‐Chloroform method (Fuhrman et al., 1988). DNA concentration was measured with the Qubit HS dsDNA Assay kit in a Qubit 2.0 Fluorometer (Life Technologies, Carlsbad, CA, USA) according to manufacturer protocols. The V4–V5 region of the 16S rRNA gene was amplified with the primers 515FB: GTGYCAGCMGCCGCGGTAA and 926R: CCGYCAATTYMTTTRAGTTT (Parada et al., 2016). Amplicons were sequenced in a MiSeq Illumina platform (2 × 300 bp). Both PCR and sequencing were performed at the Dalhousie University CGEB‐IMR, following their published protocols available at https://imr.bio/protocols.html. Sequences were deposited in the European Nucleotide Archive (ENA) under BioProject accession number PRJEB64435.

### Epilithic biofilm community analyses

Amplicon reads were analyzed using the DADA2 pipeline (Callahan et al., 2016) to characterize amplicon sequence variants (ASVs) (Callahan et al., 2017). Rarefaction curves were generated with a fixed sampling effort of 6215 reads per sample, due to the size of the smallest dataset from one replicate of the epilithic biofilm control (Appendix S1: Figure S2).

To examine the microbial beta diversity after grazing (TI) or the grazer pedal mucus effect (NTI) of the five most abundant grazer species, we used nonmetric multidimensional scaling (NMDS) ordination, based on Bray–Curtis dissimilarities. To compare the composition among treatments, we conducted a permutational ANOVA (PERMANOVA) (Anderson & Walsh, 2013). To determine which treatment differed from others, after a significant PERMANOVA, we conducted pairwise post hoc tests with false discovery rate (FDR) correction for multiple comparisons (Benjamini & Hochberg, 1995). To compare richness and Shannon diversity among treatments, we conducted a separate one‐way ANOVA or Kruskal–Wallis, respectively, after corroborating approximate homoscedasticity in both cases and large deviations from normality in the case of Shannon diversity and considered treatment (grazer species and control) as a fixed factor. We used Tukey's or Dunn's post hoc test to establish the pattern of differences.

All graphics and statistical analyses were carried out in R with the RStudio interface (Racine, 2012). Most community analyses were carried out using packages vegan v2.5‐6 (Oksanen et al., 2013) and phyloseq v1.30.0 (McMurdie & Holmes, 2013).

### Calculating the trophic and non‐trophic impact of grazers on epilithic biofilm ASVs: Interaction strength estimation and bipartite network analysis

After characterizing ASVs using the DADA2 pipeline (Callahan et al., 2016), we applied a centered log‐ratio (clr) transformation (Aitchison, 1982) for the analysis of compositional data (Gloor et al., 2017). The clr transformation uses the geometric mean of the sample vector as the reference. Then, quantification of grazer per capita effects on the relative abundance of microbial groups was estimated by the Dynamic Index (DI) (Berlow et al., 1999), Equation (1), where

(1) $$\mathrm{DI}=\frac{ln\left(\frac{N}{D}\right)}{Y_{t}}$$

ln is the natural logarithm; *N* is the relative abundance of ASVs in the treatment where grazers are present (i.e., “grazing treatments”); *D* is the relative abundance of ASVs in the treatment where grazers are absent (i.e., “Control of epilithic biofilm”); *Y*, the abundance of the grazer (i.e., our experiment used an abundance of 1 grazer per replicate); and *t*, time (i.e., our experiment only used one time). This index is recommended for short‐term experiments, comparing both positive and negative effects and when the resources exhibit a positive exponential growth (Berlow et al., 1999). We constructed bipartite networks of positive and negative TIs and NTIs between macro‐grazers and microbial ASVs. The network visualization was performed with the software Cytoscape (https://cytoscape.org). Nestedness metrics were calculated with the bipartite R package (Dormann et al., 2008). Also, specialization indices of bipartite networks (Blüthgen et al., 2006, 2007) were calculated at the network level (*H*_2_′) using H2fun and dfun functions from the bipartite R package (Dormann et al., 2008) in Software R (http://www.r-project.org). The interpretation of each metric is described in Appendix S1: Table S7.

## RESULTS

### Grazer effects on integrated measures of epilithic biofilm abundance (chlorophyll *a* and cover) after 24 h

**FIGURE 2:**
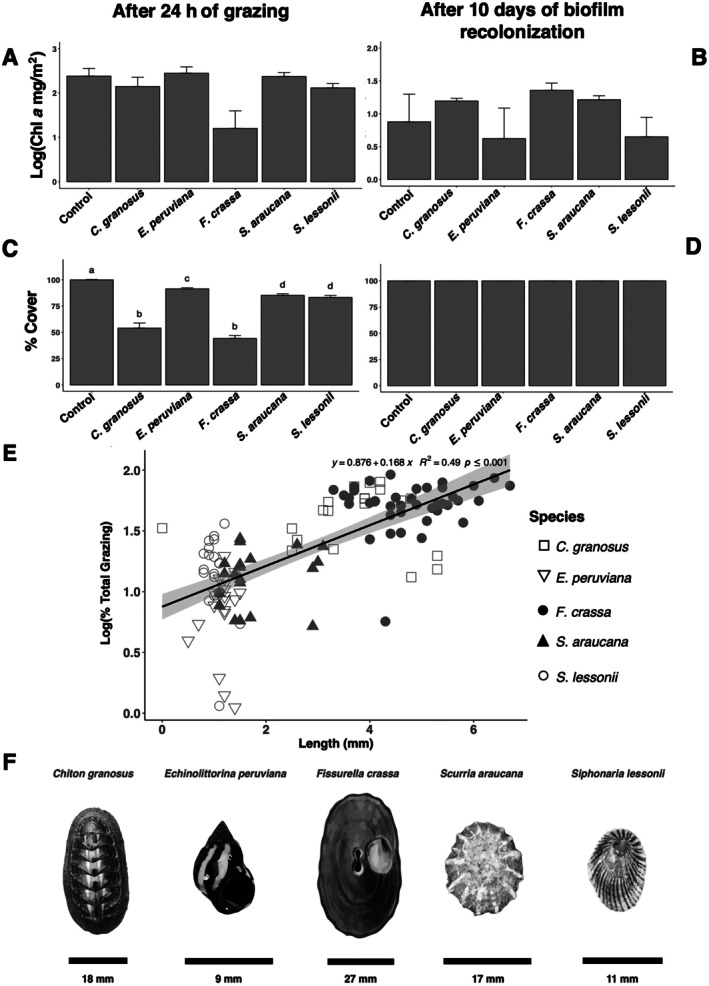
Grazing effect on epilithic biofilm. (A, B) Biofilm chlorophyll *a* content and (C, D) biofilm cover after 24 h of grazing, and biofilm recolonization over 10 days in the grazed area (mean ± SE). Different letters above bars indicate significant differences among treatments (Games‐Howell test, experiment‐wise error rate = 0.05). (E) Linear regression showing the relationship between total grazing percentage and body size (in millimeters) of *Chiton granosus* (□), *Echinolittorina peruviana* (▽), *Fissurella crassa* (●), *Scurria araucana* (▲), and *Siphonaria lessonii* (○). (F) Grazers species selected for analysis. A scale bar is provided for each species. Images credits: Ana María Valencia.

Although the chlorophyll *a* concentration (in milligrams per square meter) was lower under the *F. crassa* treatment and, to a lesser extent, under *S. lessonii* after 24 h of grazing, there were no statistical differences among the grazer species and the control group (Figure 2A, Welch's ANOVA, df = 5, *p*‐value = 0.22). The per capita effect on the chlorophyll *a* content of epilithic biofilms, as calculated using the DI (Appendix S1: Figure S3a), indicated that both *F. crassa* and *S. lessonii* had a significant impact on epilithic biofilm within a 95% CI.

In contrast, all grazer treatments had a significant negative effect on the cover of epilithic biofilms (mean% ± SE) compared to the control group (99.9 ± 0.4) (Figure 2C). However, the chiton *C. granosus* (54 ± 4.8) and the keyhole limpet *F. crassa* (44.2 ± 2.8) had more significant effects compared to *E. peruviana* (91.4 ± 1.0), *S. araucana* (85.2 ± 1.5), and *S. lessonii* (83.2 ± 1.9). The cover analysis of the same experiment revealed statistical differences among all the grazer species treatments (Figure 2C, Welch's ANOVA, df = 5, *p* < 0.0001) (Appendix S1: Table S3).

The per capita effect, calculated using the DI, yielded consistent results, with *C. granosus* and *F. crassa* having the highest impact, followed by *S. araucana* and *S. lessonii*, and finally *E. peruviana* treatments (Appendix S1: Figure S3c). Among the grazers, *F. crassa* (42.9 mm ± 3.9) was by far the largest, closely followed by *C. granosus* (34.3 mm ± 2.5). In comparison, *S. araucana* (16.1 mm ± 0.8), *E. peruviana* (10.1 mm ± 0.2), and *S. lessonii* (8.7 mm ± 0.1) were smaller in decreasing order (Appendix S1: Table S3). Given the correlation between body size and consumption rates, we compared the relationship between grazing percentage and the size of the grazer assemblage. Our analysis revealed a positive correlation between these two variables (*R* ^2^ = 0.49, *p*‐value < 0.001). Consequently, even with a different type of radula, larger grazers have a greater potential to impact the cover of epilithic biofilms (Figure 2E).

Our observations with scanning electron microscopy to investigate changes in the coverage and structure of the epilithic biofilm community, revealed a significant reduction in biomass in treatments involving *F. crassa* and *C. granosus* compared to *E. peruviana*, *S. araucana*, and *S. lessonii* (Appendix S1: Figure S4). Furthermore, when comparing the images and remaining microorganisms in the first two treatments, we observed that the Steroglossa radula of *C. granosus* destroyed all cell structures. Conversely, the Rhipidoglossa radula of *F. crassa* did not have the same effect on the remaining cells (Appendix S1: Figure S4).

### Grazer effects on integrated measures of epilithic biofilm abundance (chlorophyll *a* and cover) after 10 days of epilithic biofilm recolonization

Following the grazing experiment and a 10‐day period of biofilm recolonization, there were no significant differences observed in the chlorophyll *a* content (Figure 2B, Welch's ANOVA, df = 5, *p*‐value = 0.45) or the cover (Figure 2D) of the grazed area among the treatments. This indicates a complete recovery of the epilithic biofilm. The per capita effect, as measured by the DI, for both chlorophyll *a* content and cover was either negligible or not statistically significant in all treatment groups (Appendix S1: Figure S3b,d).

### Assessing grazer's trophic and non‐trophic effects on epilithic biofilm community: Epilithic biofilm community analyses

**FIGURE 3:**
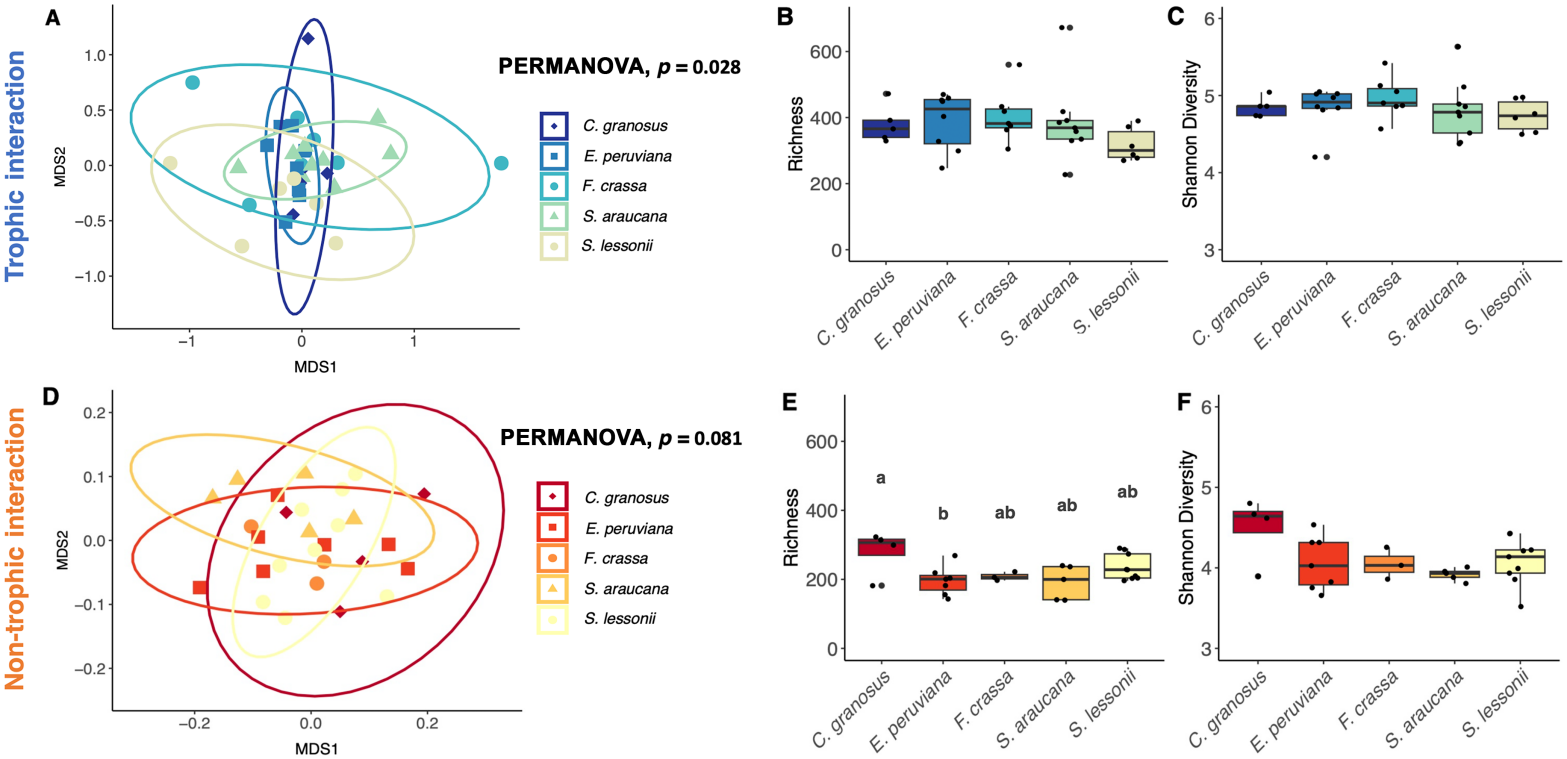
Bacterial community compositional similarity, richness, and diversity following 10 days of biofilm recolonization after trophic and non‐trophic interactions with molluscan grazers. (A, D) Nonmetric multidimensional scaling (NMDS) ordination plots based on Bray–Curtis distances. Each grazer species treatment is represented by a specific shape surrounded by a 95% CI ellipse: (♦) *Chiton granosus*, (■) *Echinolittorina peruviana*, (●) *Fissurella crassa*, (▲) *Scurria araucana*, (●) *Siphonaria lessonii*. (A) Stress = 0.186, (D) Stress = 0.192. (B, E) Mean richness, and (C, F) mean Shannon diversity index (mean ± SE). Different letters above bars indicate significant differences based on the Tukey test, experiment‐wise error rate = 0.05. PERMANOVA, permutational ANOVA.

Statistical analysis using PERMANOVA revealed significant differences in bacterial community similarity following TIs (df = 4, *p*‐value = 0.028, Figure 3A). However, after applying the FDR post hoc test for correction, the differences were no longer statistically significant (Appendix S1: Table S4). On the other hand, the composition and relative abundances of bacterial communities remained unchanged after NTIs across all five grazer treatments (PERMANOVA, df = 4, *p*‐value = 0.081, Figure 3B).

The comparison of richness and Shannon diversity index of bacterial communities following TIs with the five grazer species did not reveal statistically significant differences (Figure 3B,C; Kruskal–Wallis test, df = 4, *p*‐value = 0.288, and ANOVA, df = 4, *p*‐value = 0.684, respectively). However, microbial richness exhibited significant variation among the grazers' treatments after NTIs (Figure 3E, ANOVA, df = 4, *p*‐value = 0.033), while microbial Shannon diversity remained similar (Figure 3F, ANOVA, df = 4, *p*‐value = 0.079). Specifically, the microbial richness of the *C. granosus* and *E. peruviana* grazing treatment differed significantly (Tukey post hoc test, *p*‐value = 0.04, Appendix S1: Table S5a), with *C. granosus* (279 ± 33 richness) showing higher values compared to *E. peruviana* (196 ± 16 richness).

### Calculating the trophic and non‐trophic impact of grazers on epilithic biofilm ASVs: Interaction strength estimation and bipartite network analysis

**FIGURE 4:**
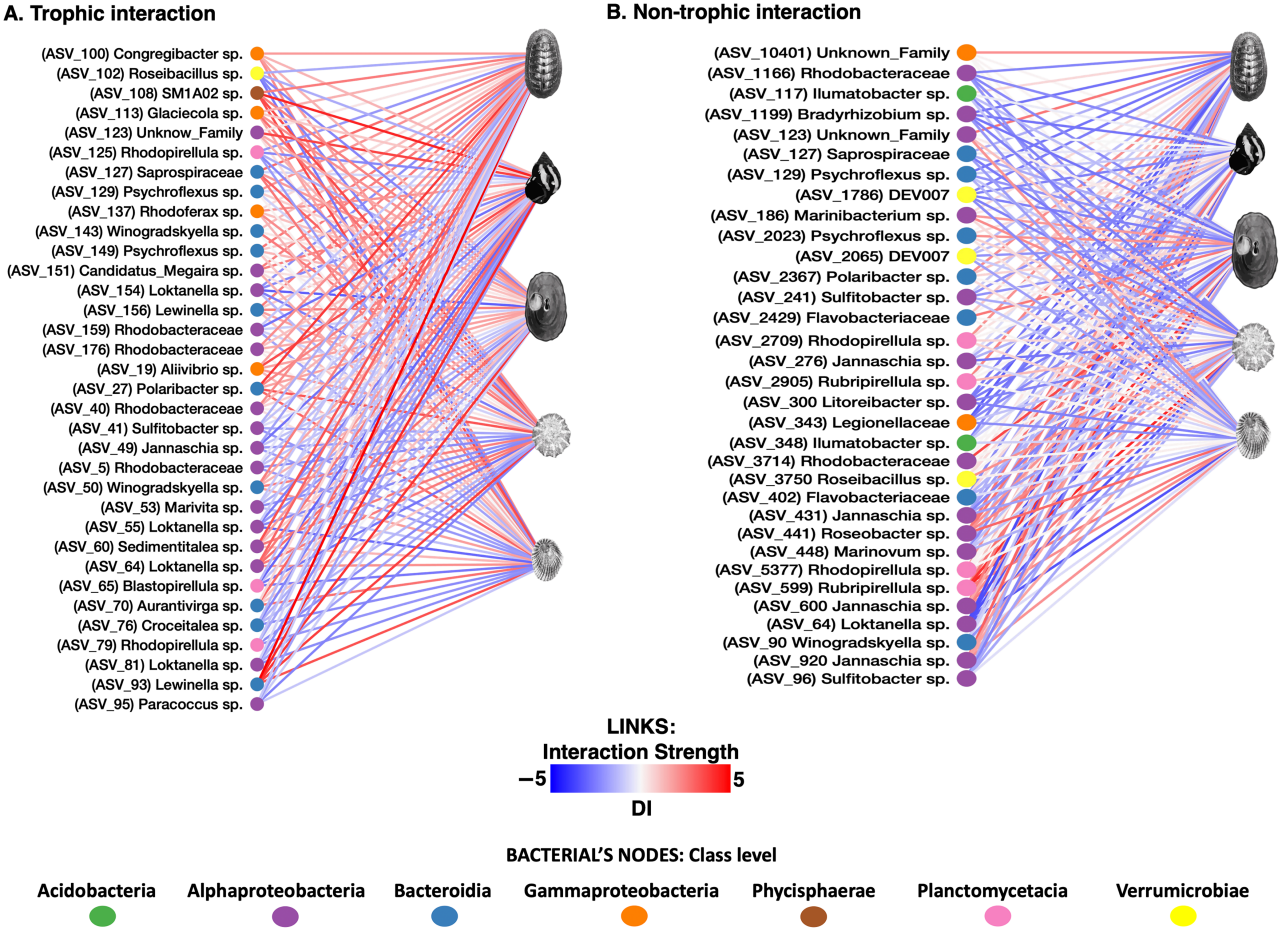
Bipartite network illustrating interactions between molluscan grazer species and microbial groups (ASVs). The node's color is the bacterial class level. The color of the links represent the sign (positive or negative) and magnitude of the interaction strength calculated using the Dynamic Index (DI). Panel (A) shows trophic interactions, while panel (B) depicts non‐trophic interactions. Image credits: Ana María Valencia.

A bipartite network for TIs and NTIs illustrating positive and negative interaction strengths was constructed (Figure 4). Following 10 days of epilithic biofilm recolonization, a total of 20 and 23 ASVs were discovered interacting trophically positively and negatively with grazers. On the other hand, 16 and 24 ASVs were found to have non‐trophic positive and negative interactions with grazers, respectively. Overall, both TIs and NTIs exhibited varying magnitudes and directions of individual per capita effects on microbial ASVs by *C. granosus*, *E. peruviana*, *F. crassa*, *S. araucana*, and *S. lessonii*, as estimated by the DI (Figure 4 and Appendix S1: Table S6). Notably, TIs demonstrated higher values of interaction strength (DI), both positive and negative.

These interaction patterns varied across bacterial families, with certain taxa benefiting from TIs while others were negatively impacted. Specifically, members of the Alteromonadaceae, Burkholderiaceae, Flavobacteriaceae, Halieaceae, Phycisphaeraceae, Rhodobacteraceae, Rickettsiaceae, Saprospiraceae, and Vibrionaceae families experienced positive trophic effects from specific grazers. In contrast, members of the Flavobacteriaceae, Pirellulaceae, Rhodobacteraceae, Rubritaleaceae, and Saprospiraceae families were negatively affected by TIs with other grazers. Some members of the Gammaproteobacteria, Flavobacteriaceae, Ilumatobacteraceae, Pirellulaceae, Rickettsiales, Rhodobacteraceae, and Rubritaleaceae families exhibited non‐trophic positive interactions with specific grazers. Meanwhile, members of the Family DEV007 (Verrucomicrobiales), Flavobacteriaceae, Ilumatobacteraceae, Legionellaceae, Rickettsiales, Rhodobacteraceae, Saprospiraceae, and Xanthobacteraceae families exhibited non‐trophic negative interactions with particular grazers.

The bacterial photosynthetic groups in our experiment belong to the phylum Cyanobacteria and potentially to the phyla Proteobacteria, Chloroflexi, and Firmicutes, since only some members of Chloroflexi (filamentous anoxygenic phototrophs, FAPs), Proteobacteria (purple bacteria, PB), and Firmicutes (heliobacteria) are phototrophic (Chew & Bryant, 2007). However, we found that Chloroflexi, Cyanobacteria, and Firmicutes had relative abundances lower than 0.01%, and they did not exhibit any positive or negative response to either TIs or NTIs with grazers. In contrast, in our study, the ASVs that remained in the bipartite networks and belong to photosynthetic groups within Proteobacteria (purple bacteria) included members of the class Alphaproteobacteria, specifically the purple non‐sulfur (PNS) bacteria from the Rhodobacter and Bradyrhizobium groups, both of which are aerobic phototrophic bacteria.

Upon constructing bipartite networks to analyze positive and negative interaction strengths between macro‐grazers and microbial ASVs using the DI, we obtained the nestedness metrics and specialization indices of bipartite networks (Figure 4 and Appendix S1: Figure S5 and Table S7).

The nestedness found in these networks is higher in NTIs (binmatnest temperature < 20), with greater nestedness values for negative interactions (binmatnest temperature = 16.7) than positive ones (binmatnest temperature = 20.1). The metrics discrepancy2 and discrepancy, which measure the degree of divergence from a completely nested matrix, displayed identical values across the networks and did not distinguish patterns between them. Nestedness metric based on overlap and decreasing fill (NODF) and NODF2 measure nestedness based on the degree of overlap between the rows and columns of the interaction matrix and the decreasing fill of these interactions. Higher NODF values indicate greater nestedness. For this metric, we found higher nestedness in TIs (NODF2 > 60) than in NTIs (NODF2 < 60), with higher values for positive interactions. The weighted‐interaction nestedness estimator (WINE) metric adjusts the nestedness measure to account for the weighted nature of interactions, with higher values indicating a more nested structure.

According to the wine metric, non‐trophic positive interactions have the highest nestedness (WINE = 44.6), followed by trophic positive interactions (WINE = 20.7). Weighted nestedness of overlap and decreasing abundance (WNODA) measures nestedness while accounting for the weights of interactions and their decreasing abundance. It combines aspects of both interaction strength and decreasing order to assess nestedness. The order of nestedness, from highest to lowest, is non‐trophic negative interaction (WNODA = 48.9), trophic positive interaction (WNODA = 47.9), trophic negative interaction (WNODA = 42.5), and finally, non‐trophic positive interaction (WNODA = 30.8). In summary, the nestedness metrics showed that non‐trophic negative interactions consistently show higher nestedness in metrics like binmatnest temperature and WNODA; non‐trophic positive interactions have the highest nestedness according to the WINE metric, and trophic positive interactions show higher nestedness in NODF, NODF2, and weighted NODF metrics. However, the non‐trophic negative interaction has higher nestedness according to multiple key metrics (binmatnest temperature and WNODA), making it the interaction type with the highest nestedness in this study.

Finally, *H*_2_′ is an index that measures the level of specialization of an entire bipartite network. Higher values of *H*_2_′, indicate increased nestedness, suggesting that ASVs affected by fewer grazers are a subset of those affected by more grazers. For trophic positive interaction (*H*_2_′ = 0.13), the low *H*_2_′ value indicates that positive TIs (such as feeding relationships) are relatively generalized. For trophic negative interaction (*H*_2_′ = 0.11), the low *H*_2_′ value for negative TIs suggests these interactions are also generalized. Species do not show a high level of selectiveness, indicating that they interact with a broad range of grazers. For non‐trophic positive interaction (*H*_2_′ = 0.31), the higher *H*_2_′ value for positive NTIs (such as mutualistic relationships) indicates a moderate level of specialization. Species are more selective in these interactions compared to TIs, showing that they interact with a more specific set of grazers than expected. For non‐trophic negative interaction (*H*_2_′ = 0.08), the very low *H*_2_′ value for negative NTIs (such as inhibition) suggests these interactions are highly generalized. Species in these interactions are not selective and interact with a wide range of grazers.

## DISCUSSION

Our results contribute toward unraveling the intricate network of interactions between macroorganisms and microorganisms in intertidal rocky shores (Liess & Hillebrand, 2004), allowing us to capture the dynamics of this complex ecological system. Our findings provide compelling evidence of TIs (consumptive effects) between grazers and epilithic biofilms, showcasing the substantial influence of these interactions on bacterial communities compared to NTIs (pedal mucus effects and exudates). It's important to highlight the significance of this last interaction. Although we don't know the frequency of pure TIs compared to animals' movements above the rocks, searching for food, escaping from predators, seeking shelter, and protecting from waves, it's crucial to distinguish between these effects. Both TIs and NTIs can be common in the rocky intertidal zone, and it's novel and important for future research to demonstrate their effects separately.

During this experiment, we demonstrated that some taxa benefit from TIs while others are negatively impacted. Specifically, members of the Alteromonadaceae, Burkholderiaceae, Flavobacteriaceae, Halieaceae, Phycisphaeraceae, Rhodobacteraceae, Rickettsiaceae, Saprospiraceae, and Vibrionaceae families experienced positive trophic effects from specific grazers. Ecologically, this has important community‐level implications, as it leads to an increase in microorganisms capable of organic matter degradation and cycling (breakdown of complex polysaccharides, biopolymers, and other organic compounds in marine environments) (Garrity et al., 2015; Krieg, 2011; Lee et al., 2008; Lenferink et al., 2024; Rosenberg et al., 2014; Shivaji & Reddy, 2014; Song et al., 2015); CO_2_ fixation (Dworkin et al., 2006a; Li et al., 2023); rhodopsin‐based photoheterotrophy (light‐stimulated energy acquisition via rhodopsins or similar photoproteins in heterotrophic bacteria) (Garrity et al., 2015; Krieg, 2011; Kwon et al., 2016; Li et al., 2023); stress resistance via carotenoid pigment production (enhanced microbial survival and tolerance under UV and oxidative stress) (Krieg, 2011; Lee et al., 2008; Song et al., 2015); iron cycling (involvement in iron redox transformations in aquatic systems) (Dworkin et al., 2006b; Lenferink et al., 2024); nitrogen cycling (denitrification with nitrate reduction and nitrous oxide production, and indirect ammonium oxidation via iron or sulfate [Feammox and Sulfammox]) (Breider et al., 2014; Dworkin et al., 2006a; Garrity et al., 2015; Krieg, 2011; Lenferink et al., 2024); facultative methylotrophy and carbon utilization flexibility (Garrity et al., 2015; Van Trappen et al., 2004); symbiosis as obligate intracellular endosymbionts (living within eukaryotic hosts, with potential transmission across trophic levels) (Lanzoni et al., 2019); bioluminescent bacteria, capable of quorum sensing, that form symbiotic relationships with aquatic organisms or act as pathogens of marine animals (Klemetsen et al., 2021); and bacterial predation (Rosenberg et al., 2014).

In contrast, members of the Flavobacteriaceae, Pirellulaceae, Rhodobacteraceae, Rubritaleaceae, and Saprospiraceae families were negatively affected by TIs with other grazers. From an ecological perspective, this shift alters community structure by reducing the abundance of microorganisms capable of aerobic anoxygenic photosynthesis (AAP, phototrophic process where light energy is captured and stored as ATP) (Zhang et al., 2019); ammonium assimilation (direct uptake of ammonium for biomass, avoiding nitrite or nitrate accumulation and enabling nitrogen removal in saline environments) (Rajeev & Cho, 2024) and participating in the global sulfur cycle in marine environments (high sulfide oxidase activity and degradation of diatom‐derived dimethylsulfoniopropionate [DMSP]) (Xu et al., 2024), potentially leading to a decline in those ecological functions. Although some microbial groups involved in organic matter degradation and cycling (Garrity et al., 2015; Krieg, 2011; Lee et al., 2008; Rosenberg et al., 2014; Wang et al., 2021), rhodopsin‐based photoheterotrophy (Garrity et al., 2015; Kwon et al., 2016); stress resistance via carotenoid pigment production (Krieg, 2011; Kwon et al., 2016); nitrogen cycling (denitrification with nitrate reduction) (Garrity et al., 2015; Krieg, 2011; Yoon et al., 2008); methylotrophy (Deng et al., 2024; Garrity et al., 2015) and bacterial predation (Rosenberg et al., 2014) were negatively affected by TIs, their functional role is preserved in the community through the positive response of other taxa with similar ecological functions.

Despite negative impacts on some taxa following the TIs, several ecological functions persist due to compensatory increases in other microbial groups. These include organic matter degradation and cycling, rhodopsin‐based photoheterotrophy, stress resistance via carotenoid pigments, nitrogen cycling (denitrification), methylotrophy, and bacterial predation. In contrast, other functions decline particularly AAP, ammonium assimilation, and sulfur cycling (including sulfide oxidase activity and the degradation of DMSP). These shifts may be attributed to the significant removal of biomass following the TI, particularly of large organisms such as diatoms. In their absence, the production of DMSP ceases, resulting in a decline of microorganisms that depend on it as a substrate. AAP may also be impacted, as the reduction in biomass increases light exposure for remaining microbes negatively affecting those lacking carotenoids for photoprotection. Relating to the nitrogen and carbon cycles, this pattern suggests an ecological shift toward a catabolism‐dominated microbial community, characterized by enhanced recycling, energy efficiency, and nitrogen loss, favoring microbial survival and stress resilience over biomass production.

Alternatively, some members of the Gammaproteobacteria, Flavobacteriaceae, Ilumatobacteraceae, Pirellulaceae, Rickettsiales, Rhodobacteraceae, and Rubritaleaceae families exhibited non‐trophic positive interactions with specific grazers. This suggests that exudates from certain grazers can trigger distinct functional responses within the microbial community. For example: organic matter degradation and cycling (Bondoso et al., 2015; Krieg, 2011; Li et al., 2015; Silva‐Solar et al., 2024; Sreya et al., 2023; Yoon et al., 2008); rhodopsin‐based photoheterotrophy (Krieg, 2011); AAP (Hwang et al., 2009; Zhang et al., 2019); nitrogen cycling (denitrification with nitrate reduction) (Garrity et al., 2005; Li et al., 2015; Yoon et al., 2008); stress resistance via carotenoid pigment production (Krieg, 2011); facultative methylotrophy (Garrity et al., 2015) and symbiosis as obligate or facultative intracellular endosymbionts (living within eukaryotic hosts) (Krieg, 2011; Lanzoni et al., 2019).

However, members of the Family DEV007 (Verrucomicrobiales), Flavobacteriaceae, Ilumatobacteraceae, Legionellaceae, Rickettsiales, Rhodobacteraceae, Saprospiraceae, and Xanthobacteraceae families exhibited non‐trophic negative interactions with particular grazers. These findings indicate that exudates released by certain grazers can diminish particular ecological functions within the microbial community, such as bacterial predation (Rosenberg et al., 2014); participation in the global sulfur cycle in marine environments (high sulfide oxidase activity and degradation of diatom‐derived DMSP) (Lee et al., 2005; Xu et al., 2024) and facultative intracellular parasites of eukaryotic cells (Dworkin et al., 2006b). Although some microbial groups involved in organic matter degradation and carbon cycling (Krieg, 2011; Lee et al., 2005; Martens et al., 2006; Romanenko et al., 2011; Rosenberg et al., 2014; Silva‐Solar et al., 2024; Wang et al., 2021); AAP (Hwang et al., 2009; Zhang et al., 2019); stress resistance via carotenoid pigment production (Krieg, 2011); nitrogen cycling (denitrification with nitrate reduction) (Garrity et al., 2015; Krieg, 2011); symbiosis as obligate intracellular endosymbionts (Krieg, 2011; Lanzoni et al., 2019); were negatively affected by NTIs, their functional role is preserved in the community through the positive response of other taxa with similar ecological functions. The ecological functions that persist in the microbial community despite negative NTIs include organic matter degradation and cycling, rhodopsin‐based photoheterotrophy, AAP, nitrogen cycling (denitrification with nitrate reduction), stress resistance via carotenoid pigment production, facultative methylotrophy, and symbiosis as obligate or facultative intracellular endosymbionts. In contrast, the ecological functions that may be reduced in the community are bacterial predation, participation in the global sulfur cycle (e.g., sulfide oxidase activity and degradation of DMSP), and facultative intracellular parasitism of eukaryotic cells.

Compared to control treatments without grazers, we found that all grazer treatments negatively affected bacteria involved in the global sulfur cycle. This decline may be attributed to grazer exudates, such as pedal mucus, which have a particularly strong impact on large organisms like diatoms. As diatom abundance decreases, DMSP production ceases, leading to a decline in bacteria that depend on DMSP as a substrate. Only one study (Arboleda‐Baena et al., 2022) has reported a negative impact of grazers on photosynthetic intertidal biofilms; the majority of previous research has focused instead on the increased growth of certain microalgae that attach to and proliferate within pedal mucus (Connor, 1986; Davies et al., 1992; Underwood & Murphy, 2008). Moreover, the observed reduction in facultative intracellular parasites during NTIs further supports the decline of large eukaryotic host cells. Therefore, we observe that both TIs and NTIs affect DMSP‐producing organisms such as diatoms, leading to a decrease in bacteria involved in the sulfur cycle. Further studies, including 18S rRNA‐gene analysis, are necessary to support this hypothesis. Finally, NTIs result in a microbial community characterized by enhanced recycling and increased energy efficiency, favoring microbial survival and stress resilience over predatory interactions.

In comparison with other studies on bioturbation in intertidal marine environments, our results are consistent with previously observed taxonomic shifts. For example, polychaete bioturbation has been shown to significantly increase the abundance of bacterial groups involved in organic matter degradation and sulfur oxidation, such as Flavobacteriaceae and Rhodobacteraceae (Fang et al., 2023), groups we also identified in our study. However, most previous studies report changes only at the phylum level. For example, one study showed that shrimp‐inhabited sediments had higher relative abundances of Proteobacteria, Gammaproteobacteria, and Acidobacteria, and lower levels of Planctomycetes (Laverock et al., 2010). Similarly, the presence of macrofauna (such as polychaete worms and mud shrimp) has been associated with the dominance of Bacteroidetes, Acidobacteria, Alphaproteobacteria, Deltaproteobacteria, Gammaproteobacteria, and Planctomycetes, while macrofauna absence correlated with a significant increase in Chloroflexi (Deng et al., 2020). While another study showed that in the presence of macrofauna, bacterial communities were predominantly composed of Proteobacteria, the relative abundances of Bacteroidetes, Alphaproteobacteria, and Verrucomicrobia were partially replaced by Deltaproteobacteria, Acidobacteria, and Chloroflexi as dissolved oxygen concentrations and redox potential decreased (Wyness et al., 2021).

In estuarine environments, crab bioturbation has been reported to increase the abundance of Proteobacteria and Bacteroidetes, while reducing Actinobacteria (Wu et al., 2021). Other studies also found that four bacterial groups (Proteobacteria, Firmicutes, Bacteroidota, and Actinobacteriota) increased in crab‐bioturbated soils, whereas seven groups (Chloroflexi, Bacteroidota, Acidobacteriota, Gemmatimonadota, Latescibacterota, Myxococcota, and Desulfobacterota) decreased (Wang et al., 2025).

Finally, a freshwater study in an intertidal zone showed that although worm bioturbation significantly stimulated biogeochemical processes at the water–sediment interface, it had only a marginally significant effect on bacterial community structure (Cariou et al., 2021), a finding that contrasts with our results.

Unlike the study on polychaete bioturbation (Fang et al., 2023), most other studies have found it difficult to identify which bacterial populations respond most strongly, either positively or negatively, to a specific bioturbator. In our study, we achieved a higher taxonomic resolution beyond the phylum level, and our experimental design enabled us to identify specific bacterial groups at the family or genus level that were positively or negatively affected during both TIs and NTIs with a specific grazer. At this finer scale, we observed both increases and decreases in members across all the phyla mentioned in previous studies. Additionally, the phyla reported by (Wang et al., 2025) were present in our study as well, but at low relative abundances.

While our findings share similarities with earlier bioturbation research, particularly regarding shifts in the abundance of certain bacterial groups and changes in the biogeochemical cycling of organic matter, our study stands out by explicitly quantifying the strength of positive and negative interactions with a specific grazer. This approach moves beyond a purely descriptive framework and advances the field by quantitatively integrating microbial interactions at the genus level into marine ecological networks.

### Grazer effects on integrated measures of epilithic biofilm abundance (chlorophyll *a* and cover)

Previous studies classified mollusk grazers into different functional groups due to their impact on algae (Aguilera & Navarrete, 2012a; Duffy et al., 2001; Lubchenco & Gaines, 1981; Steneck & Dethier, 1994) and microalgae (e.g., Bacillariophyta and Cyanobacteria) (Aguilera et al., 2013; Jenkins et al., 2001; Nicotri, 1977). The most commonly used methodology for analyzing grazer effects on epilithic biofilms (periphyton) is measuring chlorophyll *a* content as an indicator of photosynthetic group biomass (Jenkins et al., 2001; Underwood, 1984; Williams et al., 2000). However, some studies that compared periphyton chlorophyll *a* content with grazer density at different spatial and temporal scales did not show a relationship between grazing effort and microalgal abundance (Christofoletti et al., 2011; Jenkins et al., 2001; Underwood, 1984). In contrast, experiments involving grazing and herbivore exclusion have demonstrated a negative effect on periphyton chlorophyll *a* content following TIs with the entire grazer community (Williams et al., 2000), chitons over 25 days of grazing (Aguilera et al., 2013), littorinids over 40 days of grazing (Hidalgo et al., 2008), and limpets over one or two months of grazing (Valdivia et al., 2019). However, a positive effect on periphyton chlorophyll *a* content was observed in scurrinid and pulmonate limpets over 25 days of grazing (Aguilera et al., 2013). Our study found that after 24 h, the methodology for measuring chlorophyll *a* content had limitations in detecting the effects of all the grazers. Specifically, it only showed a negative per capita effect for one species, *F. crassa*. This finding could be correlated with the body size of *F. crassa*, as it is the largest grazer compared to other mollusk species in our study. Additionally, we demonstrated a positive correlation between body size and the percentage of total grazing.

In contrast, when using the epilithic biofilm cover methodology that we proposed, we observed differential effects across all grazer treatments, even within a short experiment duration. Another study utilizing image analysis techniques to assess biofilm cover on fiberglass panels found that optical density measurements were positively correlated with the total percentage cover of the biofilm (Anderson, 1995). This suggests that the optical density method is an effective approach following two and four months of grazing by periwinkles, littorinids, and limpets. In our study, we introduce a novel protocol for analyzing the cover of epilithic biofilm. This protocol utilizes natural intertidal rocks as the substrate, ensuring a more realistic and reproducible assessment (Protocols.io: https://doi.org/10.17504/protocols.io.e6nvw5epdvmk/v1) (Arboleda‐Baena, Pareja, et al., 2023).

Moreover, microscopy techniques have been employed in epilithic biofilm studies. For instance, bright field microscopy has been utilized for counting and identifying Bacillariophyta and Cyanobacteria groups (Aguilera et al., 2013), while scanning electron microscopy (SEM) has been used to qualitatively assess differences between grazed and non‐grazed areas (Nicotri, 1977; Williams et al., 2000). In our study, SEM analysis revealed how the two grazers with the highest impact on epilithic biofilm cover exhibited contrasting grazing patterns due to their radula structure (Appendix S1: Figure S4). *C. granosus*, with a Docoglossa radula, destroyed all remaining cells, whereas *F. crassa*, with a Steroglossa radula, left the remaining cells intact, potentially allowing for recolonization of the grazed area. Further microscopy studies should be conducted to assess the recolonization of grazed areas.

However, it is important to acknowledge that, although we made every effort to replicate intertidal environmental conditions as closely as possible, this was still a laboratory experiment and remains open to future improvements. We aimed to include all relevant variables that influence biofilm formation: we used the same species pool by collecting water directly from the natural habitat, maintained controlled temperature and light conditions, and employed surfaces from the same environment. Nevertheless, these surfaces may have been altered during the size‐standardization process, as we prioritized using uniform areas across all treatments and avoided those with preestablished microbial communities from the study site. However, randomizing rocks across all treatments minimized the risk of confounding effects due to undetected differences in surface smoothness among experimental units.

### Trophic and non‐trophic impact of grazers on epilithic biofilm community and ASVs: Interaction strength and bipartite network analysis

Previous studies have employed molecular tools to analyze the gut content of epilithic biofilms in a single species of littorinid and limpet grazers (Ding et al., 2018). However, these studies focused exclusively on oxygenic photoautotrophic groups. In contrast, our research enhances the resolution of bacterial communities and categorizes them based on their positive or negative interactions following 24 h of grazing. Our approach revealed that not only do grazers with a higher effect on cover and chlorophyll *a* concentration exert a proportional impact on the most abundant microorganisms, but surprisingly, even grazers with minimal effects on cover and chlorophyll *a* can also demonstrate a significant impact on the abundance of ASVs within the biofilm. In comparison with previous experiments that focused only on photosynthetic groups (such as those that measured chlorophyll *a*), it is important to highlight that the bacterial photosynthetic groups present in the microbial communities of this experiment exhibit a low abundance of Chloroflexi, Cyanobacteria, and Firmicutes which likely explains why these groups did not show a clear positive or negative response after TIs and NTIs. As a result, they are not visible in the bipartite network. However, the remaining bacterial photosynthetic groups in our experiment, the PNS bacteria from Rhodobacter and Bradyrhizobium groups, are aerobic phototrophic bacteria, but many phototrophic Proteobacteria are metabolically versatile, capable of switching from a phototrophic to a heterotrophic lifestyle in the absence of light, an adaptation known as mixotrophy. Photoheterotrophy and mixotrophy seem to be widespread in many aquatic environments, and PNS bacteria are just one of many photoheterotrophic groups using this strategy (Koblížek, 2015).

By examining the per capita grazing effects of the five most common species found in intertidal rocky shores, we constructed a high‐resolution bipartite network. This network allowed us to identify noninteracting nodes and measure the intensity of interactions between mollusk grazers and bacteria from the epilithic biofilms. These results are crucial for distinguishing the strength of interactions among specific microbial taxonomic units and gaining a better understanding of the connections between the macroscopic and microscopic worlds. Our results contribute to enhancing our understanding of the complex interactions between microorganisms and macroorganisms in marine systems, particularly in relation to biofouling (Callow & Callow, 2011; Navarrete et al., 2019) and the effective control of such processes (Arboleda‐Baena, Osiadacz, et al., 2023; Navarrete et al., 2020).

Previous studies have compared bipartite networks in various ecological contexts, including bacteria–microeukaryotes interactions (Fuhrman et al., 2015; Zheng et al., 2023), bacteria–virus interactions (Fuhrman et al., 2015; Weitz et al., 2013), symbiont–host, parasite–host, and/or predator–prey interactions in microorganisms (Bjorbækmo et al., 2020), as well as unicellular fungi–microorganisms (Moll et al., 2021), sponge–microorganisms (Thomas et al., 2016), plant–microorganism (Feng et al., 2019; Guo et al., 2009; Wang et al., 2023), and insect–microorganisms (Pechal & Benbow, 2016) interactions. However, most of these networks have been constructed using correlation‐based inference methods or the literature without analyzing the strength of interactions. What sets our bipartite network apart is experimentally demonstrating how the interaction strength between grazers and microbial taxa (ASVs) varies and distinguishes between TIs and NTIs. This differentiation allows us to observe the differential impact of grazer guilds on microscopic communities, presenting a valuable insight into the dynamics of these interactions. Nevertheless, it is crucial to recognize the inherent limitations of single‐grazer experiments. Future studies should consider conducting pairwise experiments to assess the cumulative effects of the grazer's guild on microbial communities.

That non‐trophic negative interactions (highest nestedness) unrelated to feeding, such as inhibitory or competitive interactions, are highly nested. This means there is a clear, predictable pattern where certain species consistently avoid others, and this pattern is well organized in the ecological network. Trophic positive interactions such as facilitation, on the other hand, show a high degree of nestedness, suggesting that generalist grazers interact with a wide range of ASVs, which are also preyed upon by more specialist feeders. Trophic negative interactions are networks less nested than trophic positive interactions but still show a significant degree of order. This might include negative interactions like predation that are somewhat less predictably ordered. Non‐trophic positive interactions (lowest nestedness), these interactions not related to feeding (e.g., mutualism, facilitation) are the least nested. This indicates that these beneficial interactions are more randomly distributed and less predictably ordered than other interaction types.

The application of specialized indices in bipartite networks (Blüthgen et al., 2006, 2007) revealed intriguing specialization patterns at the network level. This finding suggests that non‐trophic positive interactions may be directly influenced by the microbiota composition, as well as the chemical characteristics of the grazer's pedal mucus and other exudates. Supporting evidence for this hypothesis comes from previous research, which demonstrated significant variations in microbiota and pedal mucus chemistry among the five macro‐grazer species within the same locality (Arboleda‐Baena et al., 2022). This variation likely contributes to the observed differences in specialization within the bipartite networks, underscoring the importance of these factors in shaping the dynamics of NTIs in ecological networks. These findings highlight the complex interplay between grazers, their feeding characteristics, body size, and microbial communities, shedding light on the dynamics of TIs in these systems. Although our study aims to represent community‐wide interactions, it is important to acknowledge the limitations of our approach. The networks were constructed by individually assessing the effect of each grazer species on the microbial community, a method commonly used for constructing interaction networks of macroorganisms (Berlow et al., 1999; Case & Bender, 1981; Kareiva, 1994; Laska & Wootton, 1998; Paine, 1992). However, assessing potential interactions among grazer species under natural conditions is challenging, as these interactions often occur through behavioral antagonisms (Aguilera et al., 2019; Aguilera & Navarrete, 2012b). Consequently, this approach prevents us from fully quantifying all interactions within the grazer guild. Also, competitive effects among grazers or the combined chemical secretions of all grazers, which may influence microbial community dynamics, were not fully captured. Therefore, caution is advised when interpreting our results in natural field conditions involving multiple interacting grazer species.

Additionally, we only studied one season and one locality. Seasonal increases in phytoplankton concentration have been well‐documented in Chilean coastal environments (Aguilera et al., 2019; Aguilera & Navarrete, 2012b). Therefore, changes in the composition and diversity of microbial communities capable of colonizing the epilithic biofilm are expected to occur during seasons. Furthermore, a plethora of literature demonstrates the biogeography of microbes in the oceans, further supporting the idea of biogeography and the variability of microbial communities by locality (Sommeria‐Klein et al., 2021; Sunagawa et al., 2015). Consequently, further studies should be conducted in different seasons and localities to identify global patterns. We emphasize the importance of acknowledging this limitation upfront to ensure a nuanced understanding of the inferred ecological networks. It is important to clarify that soluble nutrients excreted by mollusks may be influencing the non‐trophic effect treatment. However, our experimental design, which included a short interaction time (24 h), feces control, low water residence time, and the use of filtered water to eliminate microorganisms from the water, was intended to isolate the effect of animal exudates (non‐trophic effect) from the direct impact of the animal on the biofilm through physical contact and grazing (trophic effect).

Remarkably, our results reveal a clear pattern where TIs predominantly result in positive effects on microbial abundance. This finding underscores the significant role that TIs play in driving the dynamics and structure of microbial populations within this ecosystem. By shedding light on these relationships, our study advances our understanding of the interconnectedness and functioning of ecological networks within intertidal rocky shores. The magnitude of both TIs and NTIs exhibited substantial variation across different grazers and microbial groups, emphasizing the intricate interactions between grazers and epilithic biofilms in marine systems. Notably, our analysis of bipartite networks revealed a higher degree of specialization in NTIs than in TIs, owing to distinct drivers such as the chemistry or microbiota of the pedal mucus specific to each grazer species.

This research complements the previous ecological network of the intertidal rocky shore of central Chile (Kéfi et al., 2015), showing that mollusk grazers interact not only trophically but also non‐trophically with epilithic biofilms. Additionally, it establishes that for future studies, three drivers that affect the assembly of microbial communities must be considered. These drivers depend on the type of interaction; for TIs, the drivers are the type of radula and body size, while for NTIs, the exudates and pedal mucus play a role. This work also shows that both TIs and NTIs shift the microbial community toward enhanced recycling, energy efficiency, and stress resilience. Grazer activity, through biomass removal and exudates like pedal mucus, reduces photosynthetic groups like diatoms, halting DMSP production and negatively impacting sulfur‐cycling bacteria and associated parasites. Finally, this study demonstrates the feasibility of integrating microbes into ecological networks, highlighting the complexity of natural systems. It marks a significant and pioneering effort in experimentally quantifying microbial interactions. The research offers valuable insights and methods for studying such interactions across diverse species, while also emphasizing the need for further investigation into their ecological implications.

## AUTHOR CONTRIBUTIONS

*Conception and design of the work*: Clara Arboleda‐Baena, Rodrigo De la Iglesia, and Sergio A. Navarrete. *Data collection*: Clara Arboleda‐Baena, Claudia Belén Pareja, and Javiera Poblete. *Data analysis and interpretation*: Clara Arboleda‐Baena, Javiera Poblete, Eric L. Berlow, Hugo Sarmento, Ramiro Logares, Rodrigo De la Iglesia, and Sergio A. Navarrete. *Drafting the article*: Clara Arboleda‐Baena, Rodrigo De la Iglesia, and Sergio A. Navarrete. *Critical revision of the article*: Clara Arboleda‐Baena, Eric L. Berlow, Hugo Sarmento, Ramiro Logares, Rodrigo De la Iglesia, and Sergio A. Navarrete. *Final approval of the version to be published*: Clara Arboleda‐Baena, Claudia Belén Pareja, Javiera Poblete, Eric L. Berlow, Hugo Sarmento, Ramiro Logares, Rodrigo De la Iglesia, and Sergio A. Navarrete. All authors agreed to be listed and have agreed on the submitted version of the manuscript.

## CONFLICT OF INTEREST STATEMENT

The authors declare no conflicts of interest.

## Supporting information


Appendix S1.

## Data Availability

Data sequences are available in the European Nucleotide Archive (ENA) under accession number PRJEB64435 at https://www.ebi.ac.uk/ena/browser/view/PRJEB64435.
