# Supplementary material for: Unifying microorganisms and macrograzers in intertidal rocky shore ecological networks
Source: Ecology. 2026 Jan 14;107(1):e70275. doi: 10.1002/ecy.70275 (PMC12800886; doi:10.1002/ecy.70275)
Supplement: Supplementary file 1 — Appendix S1. [file ECY-107-e70275-s001.pdf]

Clara Arboleda-Baena, Claudia Belén Pareja, Javiera Poblete, Eric L. Berlow, Hugo Sarmento, Ramiro Logares, Rodrigo De la Iglesia, Sergio A. Navarrete. Unifying microorganisms and macrograzers in intertidal rocky shore ecological networks. Ecology.

Appendix S1

Table S1. Grazer body size of the five most abundant species in terms of total biomass: *Chiton granosus*, *Echinolittorina peruviana*, *Fissurella crassa*, *Scurria araucana*, and *Siphonaria lessonii*.

Species	N	Mean body size (mm)	sd	se	ci
C. granosus	98	34.3	24.4	2.5	4.9
E. peruviana	400	10.1	3.3	0.2	0.3
F. crassa	55	42.9	28.9	3.9	7.8
S. araucana	90	16.1	7.7	0.8	1.6
S. lessonii	396	8.7	2.6	0.1	0.3

Figure S1. Diagram of the experiment conducted to quantify the trophic (grazing effect) and non-trophic (grazer pedal mucus effect) interactions between five of the most abundant intertidal grazers and epilithic biofilms. Photos credits: Clara Arboleda-Baena.

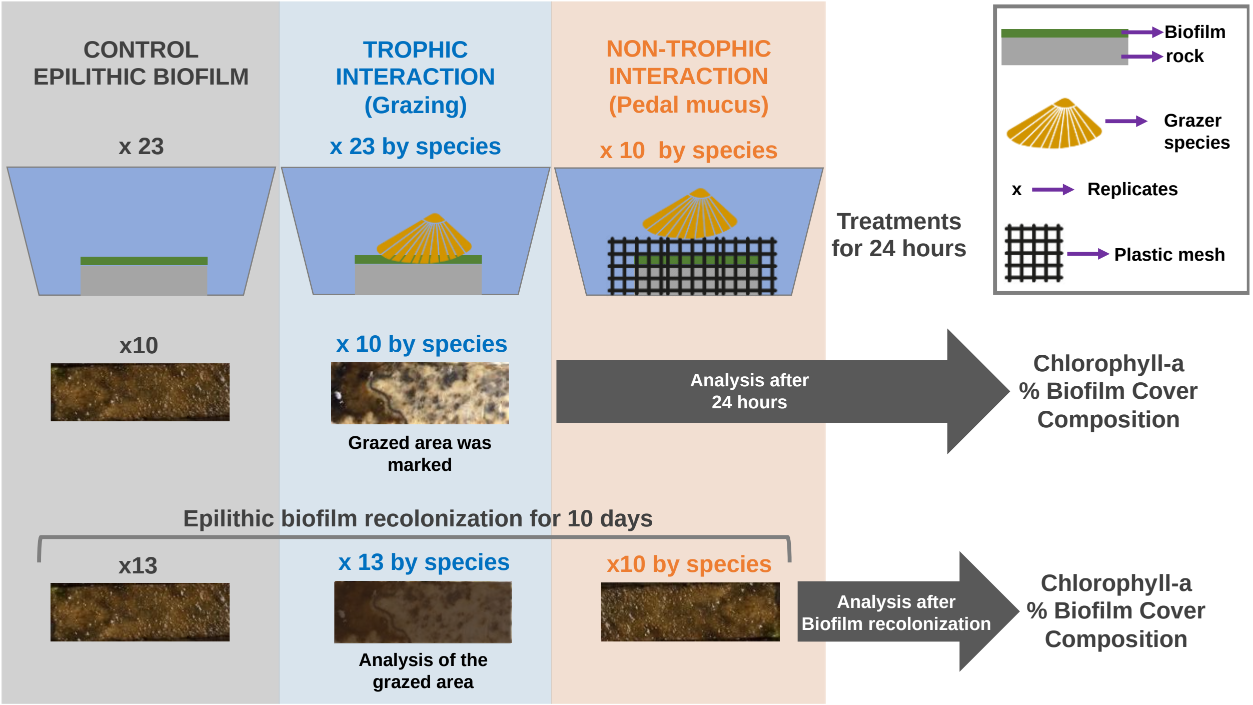

Table S2. Description, 16S rRNA sequencing characteristics of the samples.

No.	ID	Grazer	Treatment	Number of QC reads	Number of ASVs	Number of reads after filtering	Number of ASVs after filtering
1	PositiveControl_72	NA	New community Positive Control	10633	174	6215	172
2	PositiveControl_78	NA	New community Positive Control	13483	195	6215	193
3	PositiveControl_79	NA	New community Positive Control	36465	376	6215	336
4	PositiveControl_80	NA	New community Positive Control	24573	338	6215	311
5	Cg_162M	*C. granosus*	Pedal Mucus Control	12240	321	6215	312
6	Cg_166M	*C. granosus*	Pedal Mucus Control	14785	389	6215	365
7	Cg_167M	*C. granosus*	Pedal Mucus Control	8284	183	6215	182
8	Cg_169M	*C. granosus*	Pedal Mucus Control	16104	371	6215	343
9	Ep_172M	*E. peruviana*	Pedal Mucus Control	9455	287	6215	277
10	Ep_173M	*E. peruviana*	Pedal Mucus Control	7077	183	6215	183
11	Ep_174M	*E. peruviana*	Pedal Mucus Control	7838	203	6215	203
12	Ep_176M	*E. peruviana*	Pedal Mucus Control	8912	158	6215	157
13	Ep_177M	*E. peruviana*	Pedal Mucus Control	12961	212	6215	204
14	Ep_179M	*E. peruviana*	Pedal Mucus Control	7013	143	6215	143
15	Ep_180M	*E. peruviana*	Pedal Mucus Control	13637	233	6215	225
16	Fcpg_141M	*F.crassa*	Pedal Mucus Control	11518	221	6215	212
17	Fcpg_146M	*F.crassa*	Pedal Mucus Control	13764	238	6215	228
18	Fcpg_148M	*F.crassa*	Pedal Mucus Control	6215	197	6215	197
19	Sa_151M	*S. araucana*	Pedal Mucus Control	17449	267	6215	248
20	Sa_152M	*S. araucana*	Pedal Mucus Control	26433	285	6215	249
21	Sa_153M	*S. araucana*	Pedal Mucus Control	6663	141	6215	141
22	Sa_157M	*S. araucana*	Pedal Mucus Control	6820	141	6215	141
23	Sa_159M	*S. araucana*	Pedal Mucus Control	16041	226	6215	213
24	Sl_182M	*S.lessonii*	Pedal Mucus Control	16251	230	6215	214
25	Sl_183M	*S.lessonii*	Pedal Mucus Control	12183	242	6215	236
26	Sl_184M	*S.lessonii*	Pedal Mucus Control	13181	217	6215	203
27	Sl_185M	*S.lessonii*	Pedal Mucus Control	16622	225	6215	211
28	Sl_186M	*S.lessonii*	Pedal Mucus Control	23632	346	6215	305
29	Sl_187M	*S.lessonii*	Pedal Mucus Control	27171	356	6215	310
30	Sl_188M	*S.lessonii*	Pedal Mucus Control	14684	248	6215	237
31	Sl_189M	*S.lessonii*	Pedal Mucus Control	8234	205	6215	204
32	Sl_190M	*S.lessonii*	Pedal Mucus Control	7938	278	6215	277
33	Cg_101	*C. granosus*	New community of grazed rock	38590	687	6215	561

34	Cg_103	*C. granosus*	New community of grazed rock	28213	529	6215	449
35	Cg_104	*C. granosus*	New community of grazed rock	16511	398	6215	380
36	Cg_109	*C. granosus*	New community of grazed rock	8730	368	6215	364
37	Cg_110	*C. granosus*	New community of grazed rock	24743	491	6215	423
38	Ep_121	*E. peruviana*	New community of grazed rock	10210	263	6215	263
39	Ep_122	*E. peruviana*	New community of grazed rock	28927	662	6215	565
40	Ep_123	*E. peruviana*	New community of grazed rock	20056	508	6215	467
41	Ep_124	*E. peruviana*	New community of grazed rock	26654	626	6215	542
42	Ep_125	*E. peruviana*	New community of grazed rock	13408	398	6215	376
43	Ep_127	*E. peruviana*	New community of grazed rock	9580	327	6215	325
44	Ep_129	*E. peruviana*	New community of grazed rock	26492	682	6215	575
45	Ep_130	*E. peruviana*	New community of grazed rock	31844	691	6215	566
46	Fcg_82	*F.crassa large*	New community of grazed rock	11388	339	6215	332
47	Fcg_83	*F.crassa large*	New community of grazed rock	15848	439	6215	416
48	Fcg_87	*F.crassa large*	New community of grazed rock	28877	620	6215	516
49	Fcg_89	*F.crassa large*	New community of grazed rock	22996	523	6215	457
50	Fcp_113	*F.crassa small*	New community of grazed rock	15044	448	6215	424
51	Fcp_114	*F.crassa small*	New community of grazed rock	18397	635	6215	565
52	Fcp_120	*F.crassa small*	New community of grazed rock	10167	664	6215	646
53	Sa_91	*S. araucana*	New community of grazed rock	13762	387	6215	367
54	Sa_92	*S. araucana*	New community of grazed rock	21903	460	6215	420
55	Sa_93	*S. araucana*	New community of grazed rock	11404	435	6215	425
56	Sa_94	*S. araucana*	New community of grazed rock	12833	519	6215	487
57	Sa_95	*S. araucana*	New community of grazed rock	23034	850	6215	776
58	Sa_96	*S. araucana*	New community of grazed rock	22585	550	6215	497
59	Sa_97	*S. araucana*	New community of grazed rock	7691	251	6215	249
60	Sa_98	*S. araucana*	New community of grazed rock	15286	453	6215	430
61	Sa_100	*S. araucana*	New community of grazed rock	17145	396	6215	377
62	Sl_131	*S.lessonii*	New community of grazed rock	9359	344	6215	338
63	Sl_132	*S.lessonii*	New community of grazed rock	19658	499	6215	457
64	Sl_135	*S.lessonii*	New community of grazed rock	12593	329	6215	313
65	Sl_136	*S.lessonii*	New community of grazed rock	9892	309	6215	298
66	Sl_137	*S.lessonii*	New community of grazed rock	9940	427	6215	417
67	Sl_138	*S.lessonii*	New community of grazed rock	10271	332	6215	328

*During the molecular analyses, we lost 40 out of 128 samples from 11 treatments due to a poor-quality Illumina sequencing run. With an average loss of 4 samples per treatment.

Figure S2. Rarefaction curves. Sample size and ASVs are presented by treatments ■ Epilithic biofilm control, ■ Pedal mucus control, ■ Bacterial communities on grazed rock.

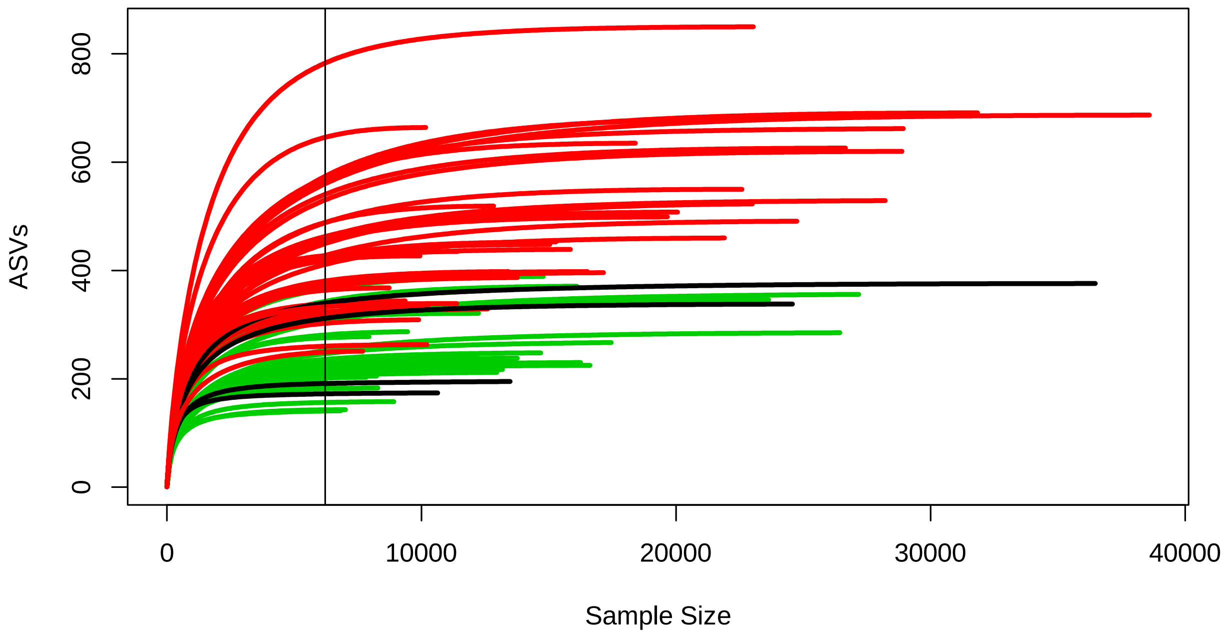

Figure S3. Interaction strength analyses with Chlorophyll-*a* content and cover after A,C) 24 hours of grazing and B,D) 10 days of epilithic biofilm recolonization of the grazed area. Bars represent 95% confidence intervals estimated through bootstrapping procedure.

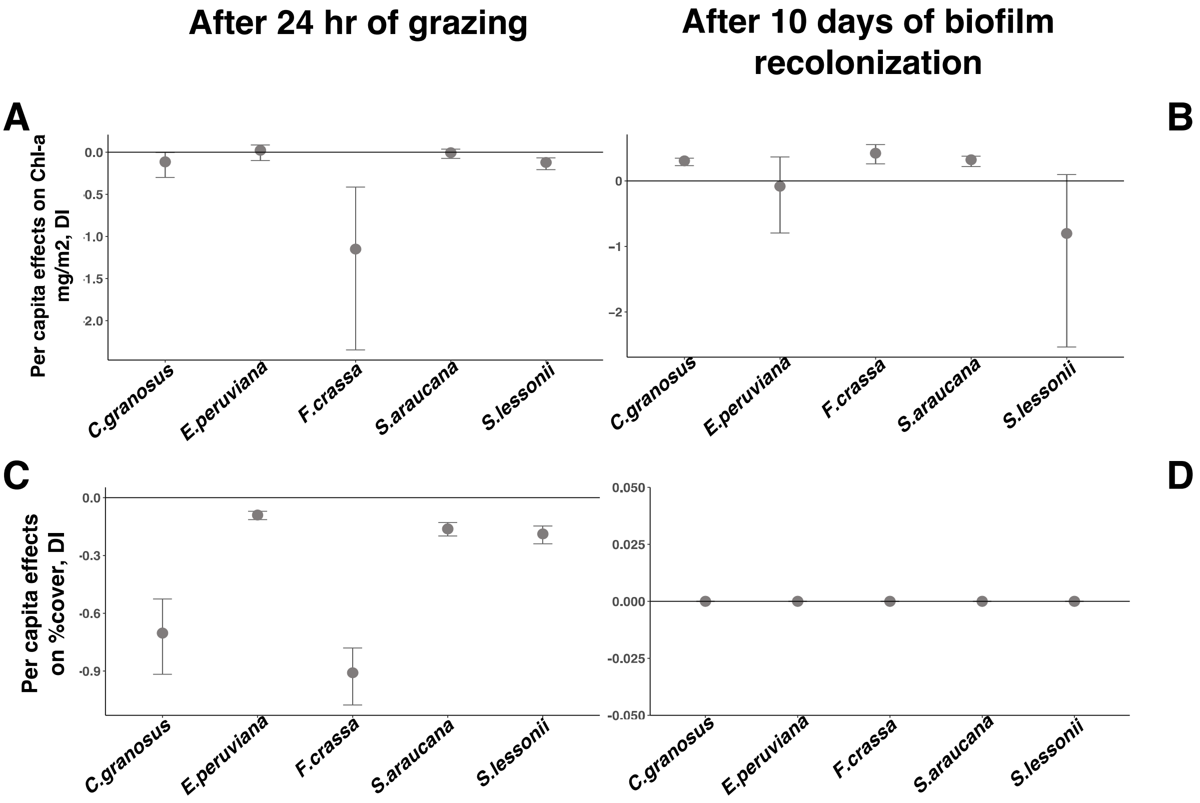

Table S3. Grazing effect over cover biofilm percentage. Games-Howell post hoc tests at the experiment-wise error rate = 0.05.

	**diff**	**ci.lo**	**ci.hi**	**t**	**df**	**p**
Control-*C. granosus*	45.89	30.66	61.12	9.504	19.3	<0.001
E. peruviana-C. granosus	37.37	21.96	52.77	7.598	20.7	<0.001
F. crassa-C. granosus	-9.77	-26.62	7.08	1.756	32.3	0.507
S. araucana-C. granosus	31.22	15.54	46.89	6.182	22.9	<0.001
S. lessonii-C. granosus	29.18	13.21	45.15	5.631	25.0	<0.001
E. peruviana-Control	-8.53	-11.94	-5.11	7.679	25.4	<0.001
F. crassa-Control	-55.67	-64.13	-47.20	19.650	40.8	<0.001
S. araucana-Control	-14.67	-19.65	-9.70	9.185	21.9	<0.001
S. lessonii-Control	-16.71	-22.89	-10.53	8.460.	20.9	<0.001
F. crassa-E. peruviana	-47.14	-55.99	-38.29	15.809	48.4	<0.001
S. araucana-E. peruviana	-6.15	-11.74	-0.56	3.326	33.1	0.024
S. lessonii-E. peruviana	-8.19	-14.84	-1.53	3.749	28.9	0.009
S. araucana-F. crassa	40.99	31.56	50.42	12.828.	55.7	<0.001
S. lessonii-F. crassa	38.95	28.93	48.97	11.457	58.0	<0.001
S. lessonii-S. araucana	-2.04	-9.46	5.38	0.826	36.2	0.961

Figure S4. Scanning electron microscopy after the grazing experiment of the five most abundant species of the intertidal rocky shore. Treatments: 1) Control biofilm without grazing, 2) Control Rock surface, grazing of 3) *C.granosus*, 4) *E.peruviana*, 5) *F.crassa* 6) *S.araucana y* 7) *S.lessonii.* Photos credits: Clara Arboleda-Baena and Claudia Belén Pareja.

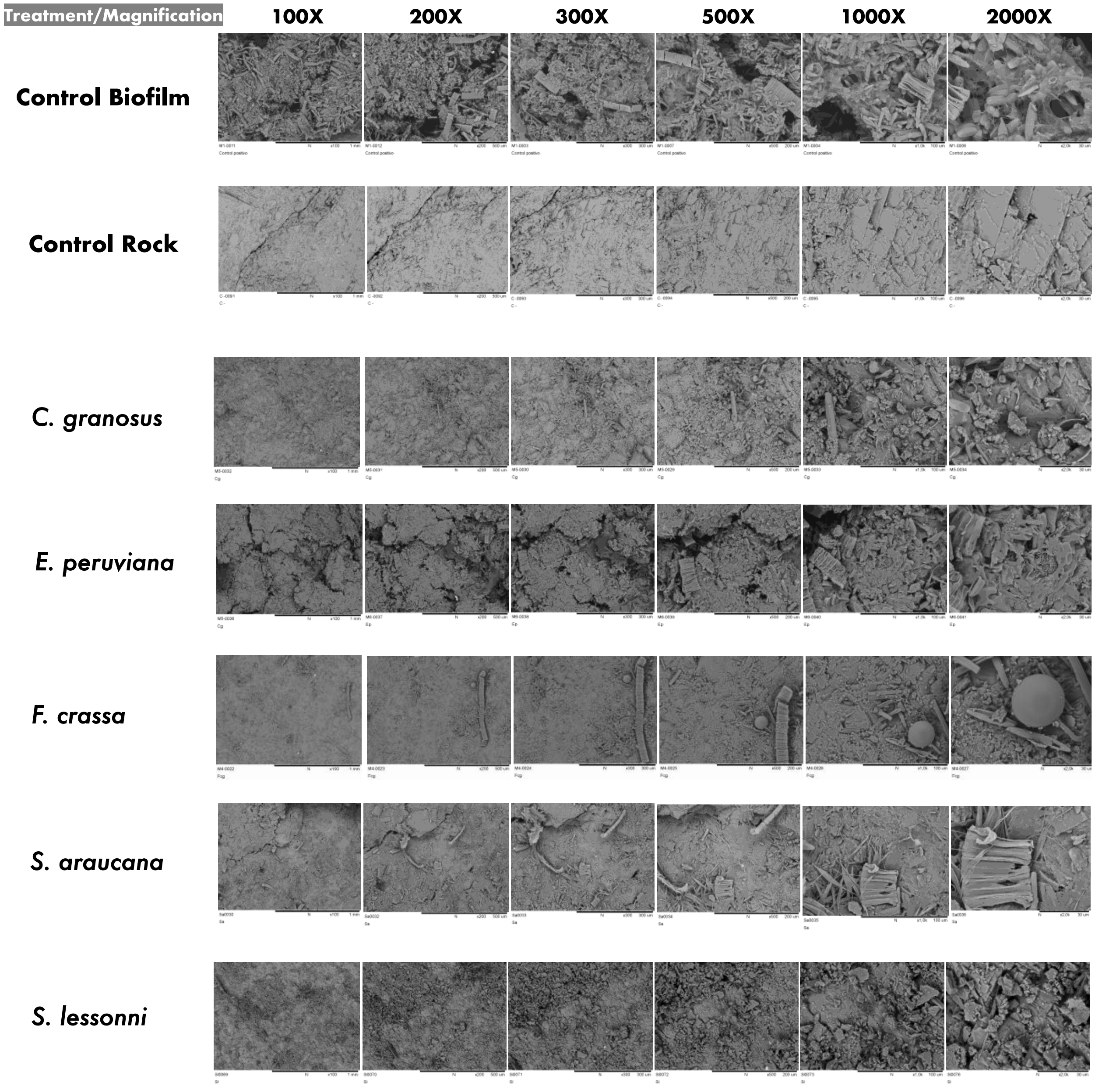

Table S4. False Discovery Rate (FDR) pairwise comparisons of bacterial communities after total, non-trophic, and trophic interaction with *C. granosus*, *E. peruviana*, *F. crassa*, *S. araucana* and *S. lessonii*. The distances used were Bray-Curtis.

a. **NON-TROPHIC INTERACTION (NTI)**

**PERMANOVA = 0.08092**

b. **TROPHIC INTERACTION (TI)**

PERMANOVA = 0.02797*

	**pairs**	**Df**	**SumsOfSqs**	**F.Model**	**R2**	**p.value**	**p.adjusted**
**1**	*S. araucana vs C. granosus*	1	0.300	1.257	0.095	0.144	0.274
**2**	*S. araucana vs F.crassa*	1	0.280	1.102	0.073	0.277	0.308
**3**	*S. araucana vs E. peruviana*	1	0.325	1.434	0.087	0.053	0.218
**4**	*S. araucana vs S.lessoni*	1	0.379	1.518	0.105	0.019	0.190
**5**	*C. granosus vs F.crassa*	1	0.309	1.193	0.107	0.185	0.274
**6**	*C. granosus vs E. peruviana*	1	0.219	0.990	0.083	0.418	0.418
**7**	*C. granosus vs S.lessoni*	1	0.291	1.151	0.113	0.219	0.274
**8**	*F.crassa vs E. peruviana*	1	0.317	1.318	0.092	0.087	0.218
**9**	*F.crassa vs S.lessoni*	1	0.344	1.277	0.104	0.066	0.218
**10**	*E. peruviana vs S.lessoni*	1	0.266	1.136	0.086	0.204	0.274

Table S5. ANOVA, KRUSKAL-WALLIS test and Tukey post hoc test multiple comparisons of bacterial communities' richness and diversity after total, non-trophic and trophic interaction with *C. granosus*, *E. peruviana, F. crassa*, *S. araucana* and *S. lessonii*. 95% family-wise confidence level.

a. NON-TROPHIC INTERACTION (NTI) Richness

ANOVA

	Df.	Sum Sq	Mean Sq	F value	Pr(>F)
Information	4	24701	6175	3.156	0.0331 *
Residuals	23	45000	1957		

TUKEY POST HOC TEST

	diff	lwr	upr	p adj
E. peruviana-C. granosus	-83.214286	-165.16809	-1.2604794	0.0453792
F. crassa-C. granosus	-71.166667	-171.03095	28.6976189	0.2511927
S. araucana-C. granosus	-87.900000	-175.61181	-0.1881928	0.0493307
S. lessonii-C. granosus	-43.833333	-122.40613	34.7394664	0.4830495
F. crassa-E. peruviana	12.047619	-78.18055	102.2757903	0.9945074
S. araucana-E. peruviana	-4.685714	-81.24686	71.8754278	0.9997409
S. lessonii-E. peruviana	39.380952	-26.51239	105.2742920	0.4157901
S. araucana-F. crassa	-16.733333	-112.22185	78.7551876	0.9846527
S. lessonii-F. crassa	27.333333	-59.83536	114.5020281	0.8835266
S. lessonii-S. araucana	44.066667	-28.86390	116.9972292	0.4050864

b. NON-TROPHIC INTERACTION (NTI) Diversity

ANOVA

	Df	Sum Sq	Mean Sq	F value	Pr(>F)
Information	4	0.8119	0.20297	2.403	0.0792.
Residuals	23	1.9430	0.08448		

c. TROPHIC INTERACTION (TI) Richness

KRUSKAL-WALLIS.
Kruskal-Wallis rank sum test
data: Richness by Information
Kruskal-Wallis chi-squared = 4.9898, *df* = 4, p-value = 0.2883

d. TROPHIC INTERACTION (TI) Diversity

ANOVA

	Df	Sum Sq	Mean Sq.	F value	Pr(>F)
Information	4	0.1894	0.04736	0.574	0.684
Residuals	30	2.4755	0.08252		

Table S6. Interaction strength (per capita effect calculated by the Dynamic Index (DI)) between macrograzers and the most abundant microbial groups.*Per capita* effect of *C.* granosus, *E. peruviana*, *F. crassa*, *S. araucana*, and *S. lessonii* during the A) Trophic interaction (grazing consumption), B) Non-trophic interaction (pedal mucus effect).

A) Trophic interaction (grazing consumption):

#	ASV	C.granosus	E.peruviana	F.crassa	S.araucana	S.lessonii
1	ASV_100	2	1	1	2	2
2	ASV_102	-2	-2	-2	-2	-3
3	ASV_108	3	4	2	3	3
4	ASV_113	1	1	2	3	2
5	ASV_123	0	4	2	1	1
6	ASV_125	-2	-2	-2	-2	-2
7	ASV_127	3	-1	2	3	3
8	ASV_129	2	-1	1	-1	0
9	ASV_137	1	2	1	2	1
10	ASV_143	1	0	0	3	0
11	ASV_149	3	1	1	0	1
12	ASV_151	2	2	0	1	1
13	ASV_154	-2	-2	-3	-2	-1
14	ASV_156	3	1	2	2	1
15	ASV_159	-1	-3	-1	-1	-2
16	ASV_176	2	1	2	0	0
17	ASV_19	2	3	2	2	1
18	ASV_27	4	2	2	3	3
19	ASV_40	2	1	1	0	1
20	ASV_41	-1	-1	-1	-2	-2
21	ASV_49	-2	-3	-1	-2	-2
22	ASV_5	0	-1	-1	-2	-2
23	ASV_50	2	2	1	2	2
24	ASV_53	-1	-1	-1	-2	-1
25	ASV_55	-1	-2	-2	-2	-3
26	ASV_60	3	2	1	3	1
27	ASV_64	3	3	3	3	2
28	ASV_65	-1	-3	-1	-3	-3
29	ASV_70	1	3	1	1	3
30	ASV_76	-1	-2	-1	-1	-2
31	ASV_79	-1	-2	0	-2	-2
32	ASV_81	-1	-2	-1	-2	-2
33	ASV_93	5	4	4	4	3
34	ASV_95	-1	-2	-1	-2	-1

B) Non-trophic interaction (pedal mucus effect):

#	ASV	C.granosus	E.peruviana	F.crassa	S.araucana	S.lessonii
1	ASV_10401	2	0	0	0	0
2	ASV_1166	-2	-2	-2	-2	-2
3	ASV_117	1	0	-1	-2	-1
4	ASV_1199	-3	-3	-3	-2	-2
5	ASV_123	3	0	0	1	2
6	ASV_127	-1	0	-2	-2	-2
7	ASV_129	-3	-1	-2	-1	-1
8	ASV_1786	-2	-2	-2	-2	-2
9	ASV_186	1	0	2	2	1
10	ASV_2023	0	1	2	1	0
11	ASV_2065	-1	-1	-2	-1	-2
12	ASV_2367	2	1	1	0	1
13	ASV_241	-1	-2	-2	-2	-1
14	ASV_2429	1	0	3	0	0
15	ASV_2709	2	0	0	1	1
16	ASV_276	-1	-1	-1	-3	-1
17	ASV_2905	2	0	0	1	0
18	ASV_300	-2	-2	-2	-2	-2
19	ASV_343	-3	-3	-2	-1	-2
20	ASV_348	-2	-1	-1	0	-1
21	ASV_3714	0	0	4	0	0
22	ASV_3750	0	1	0	2	0
23	ASV_402	-2	1	-1	-1	-1
24	ASV_431	-1	-2	-2	-2	-2
25	ASV_441	3	3	2	2	3
26	ASV_448	-1	-2	-1	-1	-1
27	ASV_5377	2	0	0	0	0
28	ASV_599	1	3	4	4	2
29	ASV_600	3	2	2	3	2
30	ASV_64	-3	-3	-3	-3	-3
31	ASV_7567	0	1	2	0	0
32	ASV_90	-1	-1	0	-2	-1
33	ASV_920	3	2	2	2	2
34	ASV_96	-1	-2	-2	-2	-1

Figure S5. Bipartite network illustrating interactions between molluscan grazer species and microbial groups (ASVs). The node's color is the class level. The color and size of the links represent the sign (positive or negative) and magnitude of the interaction strength, respectively, calculated using the Dynamic Index (DI).

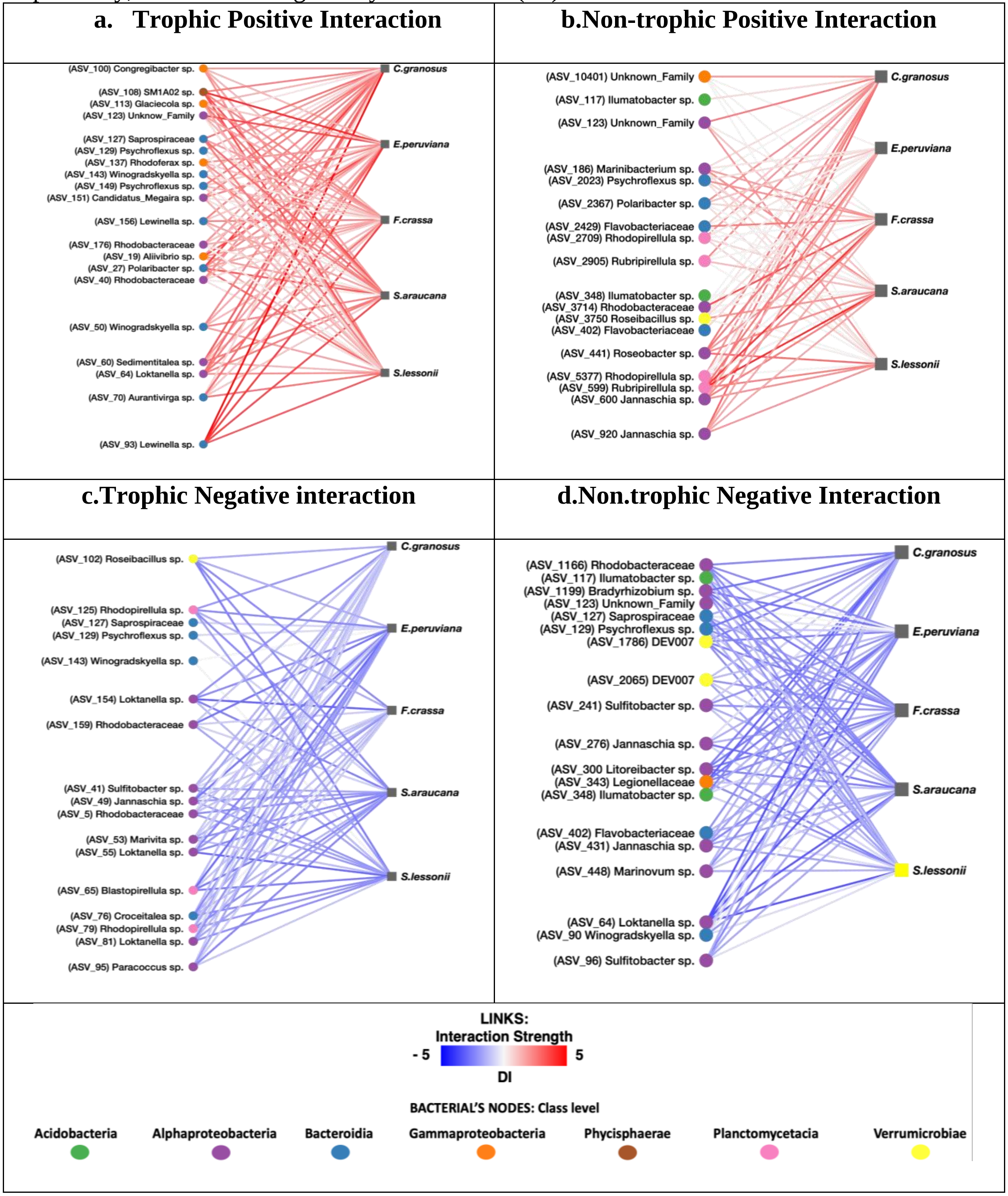

Table S7. Analysis of bipartite networks of trophic and non-trophic interactions between grazers and ASVs, including graphs and various metrics for both positive and negative interactions.

**Metric**	**TROPHIC INTERACTION**		**NON-TROPHIC INTERACTION**	
**Interaction strength**	**Positive**	**Negative**	**Positive**	**Negative**
	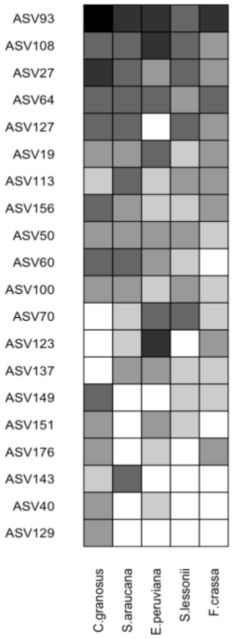	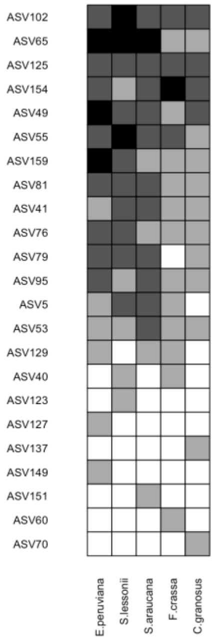	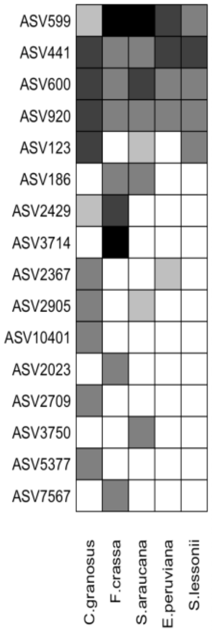	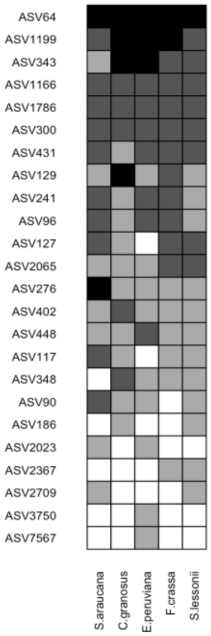
**Binmatnest temperature**	35.03582	31.29346	20.11556	16.754683
**discrepancy2**	8.00000	7.00000	8.00000	8.000000
**discrepancy**	8.00000	7.00000	8.00000	9.000000
**NODF2**	67.18958	60.36280	60.63889	58.026623
**NODF**	31.60625	21.36407	20.94658	27.544678
**weighted NODF**	33.16875	31.64607	26.84615	30.597984
**wine**	20.70314	6.73580	44.63387	4.450237
**WNODA**	47.99167	42.55862	30.76923	48.947398
**H2**	0.13	0.11	0.31	0.08
**H2min**	2.89	2.91	2.51	3.01
**H2max**	4.47	4.47	4.05	4.62
**H2uncorr**	4.26	4.30	3.58	4.48

The output from the nested function in the bipartite package provides various metrics to assess the structure of bipartite networks. Here is an explanation of each metric:

1. **binmatnest.temperature:** This metric measures the nestedness temperature of the network. A lower temperature indicates a more nested structure, where species interactions are more ordered and predictable. binmatnest calculates nestedness temperature following the function nestedtemp (0 = cold = highly nested; 100 = hot = not nested at all).
2. **discrepancy2:** This metric indicates the degree of deviation from a perfectly nested matrix. A higher value suggests more deviations and less nestedness.
3. **discrepancy:** Similar to discrepancy2, this metric measures the difference from a perfectly nested structure. The two discrepancy metrics might be calculated using slightly different methods or algorithms.
4. **NODF** is the nestedness measure proposed by Almeida-Neto and Ulrich (2011) correcting for matrix fill and matrix dimensions. Values of 0 indicate non-nestedness, those of 100 perfect nesting. NODF2 sorts the matrix before calculating the measure. NODF is closer to the version presented in the paper, while NODF2 seems to make more sense for comparisons across different networks (because it is independent of the initial presentation of the matrix). NODF calculates nestedness based on the degree of overlap between the rows and columns of the interaction matrix and the decreasing fill of these interactions. Higher NODF values indicate greater nestedness.
5. **weighted NODF:** This is the NODF metric adjusted for interaction weights. It takes into account the strength of interactions, not just their presence or absence, with higher values indicating greater nestedness.
6. **WINE (Weighted-Interaction Nestedness Estimator)**, this metric likely refers to weighted interaction nestedness estimator. It adjusts the nestedness measure to account

for the weighted nature of interactions, with higher values indicating a more nested structure. wine is one of two nestedness measure using the information on the weight of a link.

7. **WNODA (Weighted Nestedness of Overlap and Decreasing Abundance):** This metric measures nestedness while accounting for the weights of interactions and their decreasing abundance. It combines aspects of both interaction strength and decreasing order to assess nestedness.
8. **H2:** The H2'-value for the web matrix. H2' is an index describing the level of "complementarity specialisation" (or should one say: selectiveness) of an entire bipartite network (Blüthgen et al. 2006). It describes to which extent observed interactions deviate from those that would be expected given the species marginal totals. The more selective a species, the larger is H2' for the web.
9. **H2min:** Heuristic minimum H2-value for the web matrix.
10. **H2max:** Heuristic maximum H2-value for the web matrix.
11. **H2uncorr:** Uncorrected H2-values (before ranging between min and max), rounded to three digits

References

Almeida-Neto, M., and W. Ulrich. 2011. A straightforward computational approach for measuring nestedness using quantitative matrices. Environmental Modelling & Software 26(2): 173–178

Blüthgen, N., F. Menzel, and N. Blüthgen. 2006. Measuring specialization in species interaction networks. BMC Ecology 6: Article 9. https://doi.org/10.1186/1472-6785-6-9
